# Diversity in Grain, Flour, Amino Acid Composition, Protein Profiling, and Proportion of Total Flour Proteins of Different Wheat Cultivars of North India

**DOI:** 10.3389/fnut.2020.00141

**Published:** 2020-09-08

**Authors:** Raashid Ahmad Siddiqi, Tajendra Pal Singh, Monika Rani, Dalbir Singh Sogi, Mohd Akbar Bhat

**Affiliations:** ^1^Department of Food Science and Technology, Guru Nanak Dev University, Amritsar, India; ^2^Department of Food Technology, Eternal University, Baru Sahib, Himachal Pradesh, India; ^3^Multidisciplinary Research Unit, Government Medical College, Amritsar, India

**Keywords:** wheat grains, flour, protein, gluten, amino acids, solvent retention capacity, color, SDS-PAGE

## Abstract

Wheat cultivars grown at three different locations in North India were assessed for their variability in kernel and flour characteristics. Protein and the wet and dry gluten contents of the flour varied significantly (*p* ≤ 0.05) from 9.32 to 12.60%, 23.46 to 43.04%, and from 8.28 to 15.00%, respectively. Wheat varieties exhibited moderate sodium dodecyl sulfate (SDS) sedimentation and solvent retention values. Flour showed a significant (*p* ≤ 0.05) difference in the amino acid composition. Lysine, having the lowest chemical score, was the first most limiting amino acid in all wheat varieties. The variability of total flour proteins determined by SDS-PAGE showed polymorphism both in the number and intensity of bands, particularly in the molecular weight range of 35.1–42.8 kDa corresponding to the α-, β-, and γ-gliadin/low-molecular-weight glutenin subunit (LMW-GS) region. Pearson's correlation established between the various grain and flour parameters showed a significant correlation, which can result in better end product use.

## Introduction

Wheat is one of the most cultivated crops in the world and is the principal source of energy, protein, fiber, vitamins, and phytochemicals to nearly 2.5 billion people (1, 2). The global production of wheat forecasted for the year 2020 is 763 million tons (3).

Wheat grains are found in different sizes, shapes, weights, and colors. Knowledge of the physical parameters, such as size, shape, porosity, sphericity, aspect ratio, and thousand kernel wheat is important for designing equipment meant for cleaning, grading, separation, storage, transportation, packaging, and aeration and for estimating yield during milling (4, 5). Color is one of the most important quality attributes determining the consumers' acceptance of a particular food (6). The color in wheat is due to the presence of tocols, anthocyanins, and phenolic compounds, which are known to have beneficial effects on human health. The distribution of these pigments is known to vary with genotypes and different environmental conditions, thus giving rise to varied colored wheat varieties. (7).

The wheat varieties grown in India are not only numerous but also show wide diversification in the physicochemical, rheological, and functional properties (8). Kundu et al. (9) reported a significant variation in the physicochemical and flour characteristics of the same wheat varieties grown at different locations. The end product quality has been reported to be affected by the flour protein content, the wet and dry gluten contents (10), and the proportion of the different protein fractions, i.e., high-molecular-weight glutenin subunit (HMW-GS), low-molecular-weight glutenin subunit (LMW-GS), α-, γ-, and ω-gliadins, albumins, and globulins (11–14). More than 90% of the wheat cultivated in India is used for chapatti making, while the rest is used to make bread, pastries, biscuits, noodles, etc. Due to the varied genetic makeup and environmental conditions, it has become very difficult for bakers to obtain flour of consistent quality for making products.

The amino acid composition of wheat is quite unbalanced, lacking the essential amino acids like lysine, threonine, and methionine. Processing wheat into various products further depletes it of essential amino acids (15, 16). Anjum et al. (17) found that the newly released wheat varieties are nutritionally more superior than the old wheat varieties, especially in the percentages of essential amino acids, particularly lysine. Nutritional profiling of the plant foods, along with their suitability for different end product uses without compromising the quality, has become of utmost importance due to the growing trend toward organic and vegan foods. Thus, better knowledge and understanding of the various physicochemical properties, protein quality, polymorphism of the various protein fractions, and information on the amino acid composition of the varieties will be beneficial not only to plant breeders but also to processors for the manufacturing of food products without affecting their nutritional value.

To our best knowledge, none of the earlier studies has reported on the flour characteristics, protein profiling, proportion of the different protein fractions, and information on the amino acid composition together and none correlated a relation between all these parameters. This study was undertaken to evaluate the variability in the grain, flour, amino acid composition, and proportions of the different flour proteins along with their correlations.

## Materials and Methods

### Raw Material

Certified wheat varieties were procured from Punjab Agriculture University Ludhiana, Punjab, India (DBW-17, HD-2851, HD-2967, HD-3086, PBW-175, PBW-502, PBW-550, PBW-621, PBW-664, and PBW-660); Sher-e-Kashmir University of Agricultural Sciences and Technology, Srinagar, India (SW-1 and SW-2); and G.B. Pant University of Agriculture and Technology, Pantnagar, India (UP-262 and WH-1105). The wheat varieties were selected based on their different geographical locations, with geological coordinates as follows: Ludhiana (longitude 75°51′26.1972″ E, latitude 30°54′3.4740″ N, altitude 252 m.a.s.l.); Srinagar (longitude 74°47′50.5356″ E, latitude 34°5′1.1616″ N, altitude 1,587 m.a.s.l.); and Pantnagar (longitude 79°29′23.06″ E, latitude 29°1′15.74″ N, altitude 236.54 m.a.s.l.). The seeds were cleaned to remove all kinds of foreign materials like stones, dust, dirt, and broken and immature grains. The cleaned seeds from each variety were tempered with distilled water to a moisture content of 14% and left for 24 h at 4°C to equilibrate the moisture content for good bran separation. The conditioned grains were milled in a Brabender Quadrumat junior mill (Brabender OHG, Germany) to obtain white flour (72% extraction rate). The flour obtained was kept in airtight jars at −20°C until further use and thawed for 2 h at 25°C before use. All the chemicals used were of analytical grade or higher.

### Grain Characteristics

The length, width, and thickness of the wheat grains were measured using a vernier caliper with the lowest reading of 0.02 mm. The equivalent diameter (*D*_m_), sphericity, grain volume (*V*, in cubic millimeters), surface area (*S*, in square millimeters), and porosity were calculated using the relationship given by Mohsenin (18). The aspect ratio (*R*_a_) of the wheat grains was calculated as reported by Omobuwajo et al. (5). The bulk density and hundred seed volume were determined following the method of Wani et al. (4), and true density was determined using the toluene displacement method (19).

### Flour Characteristics

#### Proximate Composition

Moisture (44–15.02), protein (46–12.01), ash (08–02.01), fiber (32–10.01), and fat (30–25.01) were determined following the approved method of the American Association of Cereal Chemists (20). Carbohydrate was calculated by the difference and energy by multiplying the protein and carbohydrate by 4 and fat by 9, respectively.

#### Solvent Retention Capacity

Solvent retention capacity (SRC) was calculated according to the procedure of Ram et al. (21), with minor modifications. A flour sample (1 g) was individually suspended in 5 ml of distilled water, 5% sodium carbonate, 50% sucrose, and 5% lactic acid in a 15-ml centrifuge tube with a conical bottom and shaken vigorously for 5 s to suspend the flour. The samples were then placed horizontally on a shaking incubator (LSI-3016R, Daihan Lab Tech Co., Ltd., Namyangju, Kyonggi, South Korea) operating at 150 rpm for 20 min at room temperature to allow them to hydrate and solvate. Suspended samples were then centrifuged at 1,000 × *g* for 15 min at 25°C. The supernatant was poured off and the sample tubes were drained at a 90° angle for 15 min and weighed. The SRC values were calculated with the following formula:

$$\begin{array}{l} \text{SRC}\left(\text{g}/100\text{ g}\right)\;=\frac{\text{Wet pellet }\left(\text{g}\right)}{\text{Flour }\left(\text{g}\right)}-1\\ \;\times \frac{86}{100-\text{Flour moisture}\left(\text{g}/100\text{ g}\right)}\times 100 \end{array}$$

#### Gluten Performance Index

Gluten performance index (GPI) was determined using the SRC data following the formula of Kweon et al. (22).

$$\begin{array}{l} \text{GPI }=\frac{\text{Lactic acid SRC}}{\text{Sodium carbonate SRC }+\text{ Sucrose SRC}} \end{array}$$

#### SDS Sedimentation Value

The sodium dodecyl sulfate (SDS) sedimentation value was determined using the procedure outlined by Axford et al. (23). Five grams flour was added into a 50-ml measuring cylinder with a stopper and the contents mixed vigorously for 15 s. After 2 and 4 min, the flour contents were again thoroughly mixed for 15 s. Promptly, 50 ml of the freshly prepared SDS–lactic acid reagent (prepared by adding 2 g SDS in 100 ml of water and adding 2 ml of stock-diluted lactic acid solution, one part lactic acid plus eight parts of water by volume) was added and mixed in by inverting the cylinder four times before restarting the clock from zero again. Inversion was repeated four times at 2, 4, and 6 min before the clock was started once again from zero time. The cylinder contents were allowed to settle down for 40 min before the sedimentation values were recorded.

#### Gluten Content

Wet and dry gluten were determined as per the approved method 38-10 (20) by hand washing the dough of wheat flour (25 g) to remove the starch and other soluble components.

#### Color of Wheat Grains/Flour

The color of the wheat grains and flour was determined using UltraScan VIS Hunter Color Lab (Hunter Associates Laboratory Inc., Reston VA., USA) following the procedure of Siddiqi et al. (24). The system was calibrated before measurements using black and white tiles. Samples were taken in a glass cell, housed in a black box, and illuminated with a light source. Color readings were communicated in terms of L^*^, which indicates brightness (0–100 representing dark to whiteness); a^*^, measuring the color parameter, red to green (positive values being red and negative values being green); and b^*^, measuring the chromaticity parameter, blue to yellow (positive values being yellow and negative values being blue).

Chroma, which is the quality of color purity, intensity, or saturation of a single color, was calculated by using formula: (a^*2^ + b^*2^)^0.5^.

Hue value measures the most apparent value of color calculated by using tan^−1^(b^*^/a^*^)^2^.

The total color difference (Δ*E*) was calculated using the following formula:

Δ*E* = (dL^*^) + (da^*^) + (db^*^)^1/2^.

### Amino Acid Analysis

Samples (15 mg) of flour (defatted) were hydrolyzed in screw-capped test tubes which were dipped overnight in 2 N hydrochloric acid (HCL) to avoid any sort of contamination. Hydrolysis was carried out using 6 N HCL containing 0.1% phenol in an oven at 110 ± 1°C for 24 h. Very tight closure of the screw-capped test tubes prevents loss of HCL during the hydrolysis process. The filtrate was evaporated under vacuum at 60°C to dryness in a rotary evaporator (Buchi, Fawil, Switzerland). A suitable volume of 0.1 N HCL was added to each dried film of the hydrolyzed sample. After all the soluble materials were dissolved, the samples were filtered through a 0.22-μm filter paper (Millipore, Merck Life Science Private Limited, Mumbai, India). Amino acid analysis was performed using a Nexera Amino Acid Analyzer (Shimadzu, Kyoto Japan) equipped with pre-column derivatization. The amino acids were derivatized using mercaptopropionic acid, *o*-phthalaldehyde, and 9-fluorenylmethyl chloroformate. Chromatographic separation was carried using a Waters Spherisorb ODS2 Column (80 Å, 5 μm, 4.6 mm × 250 mm) with pH stability 2–8. The analysis was performed using 20 mmol/L phosphate (potassium) buffer (pH 6.5) as solvent A and 45:40:15 acetonitrile/methanol/water as solvent B. The separation was obtained at a flow rate of 1 ml/min using a gradient elution that allowed for 0 min at 2% B followed by a 41-min step that raised eluent B to 50%. Solvent B was then again decreased to 2% in a 43-min step for a total analysis time of 44 min. The temperature of the column oven was set at 40°C and the injection volume was 1 μl. The resolution of the amino acid derivatives was monitored through a fluorescence detector with excitation and emission set at 330 and 450 nm, respectively. Lab solutions LC/GC (Shimadzu) was used as a working station. The amino acid standard mixture was prepared by mixing 18 different amino acids (SRL, Mumbai-India) in 0.1 N HCL and included aspartic acid (Asp), threonine (Thr), serine (Ser), glutamic acid (Glu), proline (Pro), glycine (Gly), alanine (Ala), cysteine (Cys), methionine (Met), tyrosine (Tyr), arginine (Arg), phenylalanine (Phe), valine (Val), leucine (Leu), isoleucine (Ile), lysine (Lys), tryptophan (Trp), and histidine (His). The retention time of each standard in the mixture was compared with those of the digested samples and quantified by comparing the area of the sample with that of the standard mixture.

### Amino Acid Score

The amino acid score (AAS) of each flour sample was calculated by comparing the proportion of each amino acid with that of the amino acid in the reference protein, as provided by the Food and Agriculture Organization (16) based on the amino acid requirement of a child (6 months to 3 years):

$$\begin{array}{l} \text{AAS }=\frac{\text{Amino acid in test protein }\left(\text{g}\right)}{\text{Amino Acid in refrence protein }\left(\text{g}\right)}\times 100 \end{array}$$

The essential amino acids required by a child (6 months to 3 years) are histidine (2/100 g), isoleucine (3.2/100 g), leucine (6.6/100 g), lysine (5.7/100 g), methionine (2.7/100 g), phenylalanine (5.2/100 g), threonine (3.1/100 g), tryptophan (0.85/100 g), and valine (4.3/100 g).

The amino acid showing the lowest percentage was termed as the first limiting amino acid in that protein.

### SDS-PAGE of Total Flour Proteins

Defatted wheat flour (50 mg) was weighed in 1.5-ml sterile Eppendorf tubes and mixed directly with 1 ml of 2× Laemmli sample buffer (solution of pH 6.8 containing 62.5 mM Tris–HCl, 2% SDS, 25% glycerol, 0.01% bromophenol blue, and 5% ß-mercaptoethanol). The tubes were vortexed for 2 min until all the flour was completely suspended, placed and allowed to mix properly in an orbital shaker (151 rpm) for 90 min at 45°C. The tubes containing the suspended flour were heated at 100°C for 5 min and centrifuged at 12,000 × *g* for 15 min (RC 4815S, Eltek, Mumbai, India). Of the supernatant, 10 μl was loaded in each cell (Mini-Protean Tetra Cell, Bio-Rad Laboratories, Hercules, CA, USA). Proteins were separated using 4% stacking gel and 12% resolving gel. Gels were run at 25 mA until the tracking dye reached the bottom of the well, removed and stained. The gels were stained overnight with 0.1% Coomassie Brilliant Blue-R250 dissolved in 40% methanol and 10% acetic acid. Destaining was performed using 25% methanol and 10% acetic acid. The molecular weights of the polypeptides were estimated using a broad-range molecular weight standard (GeNei, Bangalore, India): myosin (205 kDa), phosphorylase B (97.4 kDa), bovine serum albumin (66.0 kDa), ovalbumin (44.0 kDa), carbonic anhydrase (29.00 kDa), soybean trypsin inhibitor (20.10 kDa), lysozyme (14.30 kDa), aprotinin (6.50 kDa), and insulin (3.50 kDa). The destained gels were quantified using a Bio-Rad EZ imager (Bio-Rad Laboratories, Hercules, CA, USA). Classification of the total flour proteins was done according to DuPont et al. (25). The band percentage of each band was determined after the intensity of all the bands in a particular lane was set at 100%. The proportion of the different flour proteins in wheat cultivars was calculated from the area of their subunits relative to the total extractable proteins. SDS-PAGE gels were run in duplicate simultaneously using aliquots of the same sample.

### Statistical Analysis

Data were expressed in terms of mean ± SD. One-way analysis of variance (ANOVA) was used and comparison of means was done using Tukey's *post-hoc* test (Minitab version 17, Minitab Inc., State College, PA, USA). Means were considered significantly different at *p* ≤ 0.05. In order to determine the relationship between the different parameters, Pearson's correlation coefficient (significance levels at *p* < 0.05 and *p* < 0.01) was performed using SPSS version 16.0 (SPSS Inc., Chicago, IL, USA).

## Results and Discussion

### Grain Characteristics

The various physical parameters of the wheat grains are given in Table 1. The moisture content of the grains varied significantly (*p* ≤ 0.05), being highest for PBW-660 (8.54%) and lowest for HD-2851 (4.81%). The grain length of the different wheat varieties varied between 6.29 and 7.30 mm and was found to be maximum for SW-2 and least for HD-2967. The breadth varied from 3.19 mm in SW to 13.55 mm in PBW-175. Thickness varied from 2.82 to 3.39 mm and was highest for HD-2851 and lowest for PBW-660 and WH-1105. The length and breadth (*L*/*B*) ratio was highest for wheat variety SW-2 (2.15) and lowest for wheat variety HD-2967 (1.78). Know-how of the length, breadth, and thickness of seeds is important in determining/regulating the aperture sizes in the machines used for the handling of grains. The length of the seed provides an indication of its natural rest position and helps in the application of compressive force, resulting in seed coat rupture with minimal damage to the kernel (5). The equivalent diameter varied significantly (*p* ≤ 0.05) and was highest for the wheat variety SW-2 (4.29 mm) and lowest for WH-1105 (4.01 mm). The equivalent diameter helps in the rough estimation of the flowing properties of the grain in the stream of air, which in turn helps during cleaning in the separation of foreign materials by pneumatic means (5).

**Table 1:** Physical characteristics of the different wheat varieties of North-India.

**Variety**	**Moisture (%)**	**Thousand kernel weight (g)**	**Hundred seed volume (g)**	**Length (mm)**	**Breadth (mm)**	**Thickness (mm)**	***L*/*B* ratio**	**Equiv. diameter (mm)**	**Sphericity (%)**	**Aspect ratio**	**Seed volume (mm^**3**^)**	**Surface area (mm^**2**^)**	**Bulk density (g/ml)**	**True density (g/ml)**	**Porosity (%)**
DBW-17	5.77 ± 0.12^DE^	40.98 ± 0.72^CD^	3.63 ± 0.15^BC^	6.66 ± 0.02^DEF^	3.49 ± 0.09^A^	2.92 ± 0.06^CD^	1.91 ± 0.04^BC^	4.08 ± 0.06^AB^	61.25 ± 0.77^ABC^	0.52 ± 0.12^AB^	22.91 ± 0.92^AB^	43.88 ± 1.18^AB^	0.82 ± 0.01^ABC^	1.16 ± 0.06^CDE^	29.10 ± 3.67^BCD^
HD-2851	4.81 ± 0.99^E^	40.17 ± 0.89^CD^	3.00 ± 0.00^D^	6.51 ± 0.09^EF^	3.35 ± 0.10^AB^	3.39 ± 0.06^A^	1.94 ± 0.08^B^	4.20 ± 0.02^AB^	64.53 ± 0.90^A^	0.52 ± 0.22^BC^	25.17 ± 0.37^AB^	46.52 ± 0.47^AB^	0.85 ± 0.01^A^	1.34 ± 0.03^ABC^	36.71 ± 1.10^ABC^
HD-2967	6.27 ± 0.17^BCDE^	40.18 ± 1.47^CD^	4.10 ± 0.17^A^	6.29 ± 0.13^F^	3.53 ± 0.08^A^	3.03 ± 0.03^ABCD^	1.78 ± 0.03^C^	4.07 ± 0.07^AB^	64.67 ± 0.66^A^	0.56 ± 0.01^A^	23.17 ± 1.00^AB^	43.73 ± 1.41^AB^	0.77 ± 0.03^E^	1.20 ± 0.06^BCDE^	35.93 ± 3.44^ABC^
HD-3086	5.71 ± 0.66^DE^	42.75 ± 0.99^BCD^	3.93 ± 0.12^AB^	6.99 ± 0.03^ABCD^	3.41 ± 0.07^AB^	2.90 ± 0.08^CD^	2.05 ± 0.05^AB^	4.10 ± 0.04^AB^	58.73 ± 0.69^C^	0.49 ± 0.01^BCD^	23.06 ± 0.66^AB^	44.58 ± 0.81^AB^	0.83 ± 0.02^ABC^	1.06 ± 0.02^E^	22.23 ± 2.66^D^
PBW-175	5.95 ± 1.09^DE^	47.24 ± 1.05^A^	4.07 ± 0.12^A^	7.25 ± 0.31^AB^	3.55 ± 0.12^A^	3.01 ± 0.03^BCD^	2.05 ± 0.07 ^AB^	4.26 ± 0.12^AB^	58.79 ± 3.33^C^	0.49 ± 0.02^BCD^	25.51 ± 2.20^AB^	47.99 ± 2.58^AB^	0.82 ± 0.01^ABC^	1.14 ± 0.15^DE^	26.82 ± 8.51^CD^
PBW-502	5.98 ± 0.91^DE^	44.00 ± 2.64^ABC^	3.50 ± 0.00^C^	7.00 ± 0.07^ABCD^	3.45 ± 0.03^AB^	2.93 ± 0.03^CD^	2.03 ± 0.01^AB^	4.14 ± 0.04^AB^	59.06 ± 0.32^C^	0.49 ± 0.00^BCD^	23.58 ± 0.54^AB^	45.24 ± 0.77^AB^	0.82 ± 0.01^ABCD^	1.27 ± 0.08^ABCD^	35.47 ± 3.45^ABC^
PBW-550	6.26 ± 0.52^BCDE^	42.49 ± 1.42^BCD^	3.97 ± 0.06^AB^	7.14 ± 0.04^ABC^	3.47 ± 0.07^AB^	2.91 ± 0.07^CD^	2.06 ± 0.04^AB^	4.16 ± 0.06^AB^	58.30 ± 0.59^C^	0.49 ± 0.01^BCD^	23.89 ± 0.97^AB^	45.83 ± 1.25^AB^	0.84 ± 0.02^AB^	1.28 ± 0.06^ABCD^	34.12 ± 2.87^ABC^
PBW-621	6.07 ± 0.54^CDE^	35.44 ± 1.07^EF^	3.60 ± 0.17^BC^	6.70 ± 0.04^DEF^	3.38 ± 0.08^AB^	2.93 ± 0.05^CD^	1.99 ± 0.04^B^	4.05 ± 0.04^AB^	60.41 ± 0.30^BC^	0.50 ± 0.01^BC^	22.38 ± 0.63^AB^	43.29 ± 0.85^AB^	0.78 ± 0.02^DE^	1.14 ± 0.08^DE^	30.92 ± 3.69^ABCD^
PBW-644	7.11 ± 0.44^ABCD^	39.71 ± 1.64^D^	3.73 ± 0.21^ABC^	6.99 ± 0.25^ABCD^	3.39 ± 0.13^AB^	3.02 ± 0.07^ABCD^	2.06 ± 0.03^AB^	4.15 ± 0.12^AB^	59.44 ± 0.93^C^	0.49 ± 0.01^CD^	23.89 ± 1.88^AB^	45.58 ± 2.66^AB^	0.79 ± 0.00^CDE^	1.27 ± 0.05^ABCD^	37.56 ± 2.52^ABC^
PBW-660	8.54 ± 0.47^A^	40.28 ± 0.67^CD^	3.83 ± 0.29^ABC^	6.80 ± 0.12^CDE^	3.37 ± 0.06^AB^	2.82 ± 0.11^D^	2.02 ± 0.01^AB^	4.02 ± 0.10^B^	59.04 ± 0.38^C^	0.50 ± 0.00^BCD^	21.77 ± 1.50^B^	42.65 ± 2.03^B^	0.81 ± 0.01^BCDE^	1.13 ± 0.08^DE^	28.58 ± 5.65^BCD^
SW-1	7.82 ± 0.36^ABC^	33.05 ± 2.19^F^	3.00 ± 0.00^D^	6.45 ± 0.07^EF^	3.19 ± 0.08^B^	3.27 ± 0.15^ABC^	2.02 ± 0.06^AB^	4.07 ± 0.10^AB^	63.06 ± 0.83^AB^	0.49 ± 0.02^BCD^	22.94 ± 1.32^AB^	43.63 ± 1.74^AB^	0.85 ± 0.01^A^	1.36 ± 0.07^AB^	37.32 ± 2.75^ABC^
SW-2	6.16 ± 0.63^CDE^	45.58 ± 1.46^AB^	4.00 ± 0.00^AB^	7.30 ± 0.06^A^	3.39 ± 0.09^AB^	3.20 ± 0.20^ABC^	2.15 ± 0.06^A^	4.29 ± 0.05^A^	58.80 ± 1.15^C^	0.46 ± 0.01^D^	26.03 ± 1.01^A^	48.69 ± 1.15^A^	0.84 ± 0.01^AB^	1.43 ± 0.03^AB^	41.11 ± 0.90^A^
UP-262	7.98 ± 0.29^AB^	39.68 ± 0.57^D^	3.00 ± 0.00^D^	6.57 ± 0.04^EF^	3.32 ± 0.09^AB^	3.32 ± 0.12^AB^	1.98 ± 0.05^B^	4.17 ± 0.09^AB^	63.48 ± 1.33^AB^	0.51 ± 0.01^BC^	24.62 ± 1.55^AB^	45.91 ± 1.94^AB^	0.82 ± 0.01^ABC^	1.34 ± 0.05^ABC^	38.67 ± 1.85^AB^
WH-1105	7.88 ± 0.38^ABC^	39.12 ± 1.12^DE^	3.87 ± 0.15^ABC^	6.85 ± 0.21^BCDE^	3.35 ± 0.20^AB^	2.82 ± 0.13^D^	2.05 ± 0.06^AB^	4.01 ± 0.18^B^	58.61 ± 0.84^C^	0.49 ± 0.01^BCD^	21.75 ± 2.72^B^	42.66 ± 3.77^B^	0.83 ± 0.00^AB^	1.22 ± 0.01^BCDE^	31.78 ± 0.64^ABCD^

*Data are presented as the mean ± SD. Means with different superscripts in columns differ significantly (p ≤ 0.05) among varieties and means with similar superscripts differ non-significantly (p ≥ 0.05). n = 3*.

Shape is important for the separation of foreign materials, grading, quality evaluation, and heat and mass transfer calculations. The shape is usually expressed in terms of sphericity and aspect ratio (4) for particular food materials. Sphericity measures how much resemblance an object has to that of a perfect sphere of the same volume. The sphericity of the wheat varieties varied significantly and ranged from 58.30 to 64.67%. Wheat variety PBW-550 showed the lowest value for sphericity; the highest value was observed in HD-2967. The sphericity values of the wheat seeds indicate that these seeds are more elongated and may slide rather than roll out, which becomes an important factor for the design of hoppers, dehullers (5), and other processing instruments. The aspect ratio relates the width to the length and is an indication of the oblong shape of the seed (5). Among the wheat varieties, HD-2967 had the highest aspect ratio of 0.56 and SW-2 had the lowest value of 0.46. The seed volume and surface area were observed in ranges of 21.75–26.03 and 42.65–48.69 mm^2^, respectively, and varied significantly (*p* ≤ 0.05) among the wheat cultivars. SW-2 had the highest and WH-1105 had the lowest value for seed volume. The surface area was highest for SW-2 and lowest for PBW-660.

The bulk and true density of the wheat varieties varied from 0.77 to 0.85 g/ml and from 1.06 to 1.43 g/ml, respectively (Table 1). Significant (*p* ≤ 0.05) differences were observed in the bulk and true density of the wheat varieties. The highest bulk density was observed in wheat varieties HD-2851 and SW-1 and lowest in HD-2967. Among the wheat varieties, SW-2 showed the highest and HD-3086 showed the lowest value of true density. Knowledge of the density of the seeds is useful in estimating the yield of the product, quality, output from machines, and also in the transportation of seeds (4, 5).

Porosity values are helpful in the packaging, storage, aeration, and in the system design of various mass and heat transfer processes like drying, frying, heating, baking, extrusion, and cooling (4, 6). The porosity of the wheat varieties ranged from 22.23 to 41.11%, which showed a significant (*p* ≤ 0.05) difference and was highest for SW-2 (41.11%) and lowest for HD-3086 (22.23%). High porosity values are associated with better aeration and high water vapor permeability during processes like drying, frying, heating, baking, extrusion, and cooling (26).

Hundred seed volume varied significantly (*p* ≤ 0.05) from 3.00 to 4.10 ml among the cultivars (Table 1). HD-2967 showed the highest whereas SW-1 and UP-262 showed the lowest value for hundred seed volume. Thousand kernel weight (TKW) showed a significant (*p* ≤ 0.05) difference among the wheat cultivars. TKW ranged from 33.05 g for SW1 and 47.24 g for wheat variety PBW-175. TKW is important for estimating the milling yield and grain quality; longer, round, and sound grains have higher TKW. The wheat grains used in this study were from a small to a large category based on the classification of Williams et al. (27), who classified wheat kernels based on grain weight (TKW) as 15–25 g (very small), 26–35 g (small), 36–45 g (medium), 46–55 g (large), and over 55 g (very large).

Earlier, Baljeet et al. (28) reported on the lengths of Indian wheat varieties in the range of 6.47–7.07 mm, widths 3.0–3.50 mm, thicknesses 2.10–2.60 mm, *L*/*B* ratios 2.02–2.15, equivalent diameters 3.50–4.00 mm, sphericity 53.59–60.14%, surface areas 31.46–40.69 mm^2^, grain volumes 23.26–34.26 mm^3^, porosity 33.52–39.54%, and thousand kernel weights of 34.81–47.47 g. Our results are in close proximity to those of the reported study; however, the slight variations between the two studies might be due to differences in the cultivars used, and to some extent, the agronomic practices followed.

### Grain Color

Grain color of wheat varieties evaluated in terms of CIE (International Commission on Illumination) color values (L^*^, a^*^, and b^*^) showed varietal difference and is summarized in Table 2. L^*^, which indicates lightness (0 is black and 100 is white), was observed in the range of 57.43–61.38. Statistical analysis revealed a significant (*p* ≤ 0.05) difference in L^*^ among the wheat cultivars. The highest L^*^ was observed in wheat cultivar HD-2967 and the lowest in HD-2851. The higher L^*^ of HD-2967 indicated its lighter color as compared to the other wheat varieties. The CIE a^*^ values ranged from 6.06 to 7.26 and varied significantly (*p* ≤ 0.05) among the different cultivars. Wheat variety PBW-660 showed the highest value for a^*^, and the lowest value was observed in WH-1105. Positive a^*^ values indicate a red tint among the different wheat varieties. The b^*^ value was highest in the wheat variety PBW-502 (20.24) and lowest for HD-2851 (17.78), thus giving an indication of a more yellow color. The chroma values showed a similar pattern to the b^*^ values and varied significantly (*p* ≤ 0.05) from 18.82 to 21.37. Hue values showed a significant (*p* ≤ 0.05) difference among the different wheat cultivars and ranged from 69.49 (for SW-1) to 72.57 (for UP-262). Previous studies have reported L^*^ values of 35.2–58.9, a^*^ of 1.2–10.1, b^*^ of 11.5–27.4, chroma of 12.6–28.6, and hue values of 58.8–85.0 for wheat cultivars (29). Δ*E*, which measures the total color difference, varied from 60.43 to 64.89 and followed the same pattern as L^*^.

**Table 2:** CIE color parameters of the grains and flours of the different wheat varieties of North-India.

**Variety**	**Wheat kernel**						**Wheat flour**					
	L*	a*	b*	**Δ*E***	**Chroma**	**Hue**	L*	a*	b*	**Δ*E***	**Hue**	**Chroma**
DBW-17	58.82 ± 1.19^ABC^	6.67 ± 0.11^BCD^	19.50 ± 0.38^ABC^	62.32 ± 1.25^ABC^	20.61 ± 0.39^ABC^	71.11 ± 0.13^BC^	90.82 ± 0.22^C^	0.48 ± 0.03^BCD^	10.52 ± 0.18^A^	91.32 ± 0.30^C^	87.39 ± 0.12^EF^	10.53 ± 0.18^A^
HD-2851	57.43 ± 0.37^C^	6.17 ± 0.19^CDE^	17.78 ± 0.14^D^	60.43 ± 0.39^C^	18.82 ± 0.16^D^	70.86 ± 0.51^BCD^	92.35 ± 0.14^AB^	0.22 ± 0.04^G^	8.49 ± 0.16^G^	92.74 ± 0.13^AB^	88.54 ± 0.21^A^	8.49 ± 0.16^G^
HD-2967	61.38 ± 1.82^A^	6.80 ± 0.20^AB^	19.92 ± 0.86^AB^	64.89 ± 1.98^A^	21.05 ± 0.82^AB^	71.12 ± 0.88^BC^	91.72 ± 0.27^BC^	0.47 ± 0.03^BCD^	9.64 ± 0.11^C^	92.22 ± 0.26^ABC^	87.19 ± 0.16^EFG^	9.66 ± 0.11^C^
HD-3086	58.34 ± 0.59^BC^	6.68 ± 0.24^BCD^	18.73 ± 0.39^BCD^	61.63 ± 0.56^BC^	19.88 ± 0.44^BCD^	70.38 ± 0.37^CD^	92.59 ± 1.01^AB^	0.28 ± 0.05^FG^	7.71 ± 0.07^H^	92.90 ± 1.01^AB^	87.94 ± 0.36^BCD^	7.71 ± 0.07^H^
PBW-175	59.56 ± 1.03^ABC^	6.56 ± 0.09^BCDE^	18.84 ± 0.27^BCD^	62.82 ± 1.05^ABC^	19.95 ± 0.23^BCD^	70.79 ± 0.43^BCD^	91.70 ± 0.09^BC^	0.41 ± 0.04^CDE^	9.38 ± 0.14^CD^	92.18 ± 0.08^BC^	87.52 ± 0.19^DEF^	9.39 ± 0.15^CD^
PBW-502	60.87 ± 0.98^AB^	6.85 ± 0.29^AB^	20.24 ± 0.34^A^	64.51 ± 0.80^AB^	21.37 ± 0.41^A^	71.32 ± 0.47^ABC^	92.88 ± 0.06^A^	0.28 ± 0.01^FG^	8.65 ± 0.12^FG^	93.29 ± 0.08^A^	88.15 ± 0.07^ABC^	8.65 ± 0.12^FG^
PBW-550	59.82 ± 0.99^ABC^	6.61 ± 0.09^BCDE^	19.48 ± 0.41^ABC^	63.25 ± 1.06^ABC^	20.57 ± 0.42^ABC^	71.26 ± 0.16^ABC^	92.14 ± 0.58^AB^	0.40 ± 0.03^DE^	8.74 ± 0.08^FG^	92.56 ± 0.57^AB^	87.40 ± 0.17^DEF^	8.75 ± 0.08^FG^
PBW-621	59.66 ± 0.94^ABC^	6.71 ± 0.22^ABC^	19.24 ± 0.45^ABC^	63.05 ± 1.03^ABC^	20.38 ± 0.50^ABC^	70.79 ± 0.18^BCD^	92.02 ± 0.24^AB^	0.54 ± 0.02^AB^	9.20 ± 0.06^DE^	92.48 ± 0.24^AB^	86.66 ± 0.11^GH^	9.22 ± 0.06^DE^
PBW-644	60.23 ± 0.95^ABC^	6.45 ± 0.24^BCDE^	18.91 ± 0.56^ABCD^	63.45 ± 1.08^AB^	19.98 ± 0.60^ABCD^	71.16 ± 0.32^BC^	91.70 ± 0.14^BC^	0.46 ± 0.02^BCD^	8.92 ± 0.03^EF^	92.14 ± 0.15^BC^	87.05 ± 0.12^FGH^	8.93 ± 0.03^EF^
PBW-660	59.41 ± 0.48^ABC^	7.26 ± 0.13^A^	19.65 ± 0.18^ABC^	63.00 ± 0.52^ABC^	20.95 ± 0.21^ABC^	69.72 ± 0.20^D^	92.21 ± 0.10^AB^	0.49 ± 0.01^ABC^	10.11 ± 0.06^B^	92.74 ± 0.08^AB^	87.21 ± 0.04^EFG^	10.13 ± 0.06^B^
SW-1	58.36 ± 0.65^BC^	6.93 ± 0.15^AB^	18.52 ± 0.30^CD^	61.62 ± 0.69^BC^	19.77 ± 0.26^BCD^	69.49 ± 0.62^D^	92.23 ± 0.35^AB^	0.32 ± 0.03^EF^	10.80 ± 0.08^A^	92.86 ± 0.35^AB^	88.30 ± 0.16^AB^	10.80 ± 0.08^A^
SW-2	59.20 ± 0.62^ABC^	6.66 ± 0.16^BCD^	18.49 ± 0.26^CD^	62.38 ± 0.66^ABC^	19.66 ± 0.21^BCD^	70.19 ± 0.66^CD^	91.77 ± 0.21^BC^	0.40 ± 0.03^DE^	9.55 ± 0.11^C^	92.27 ± 0.20^ABC^	87.60 ± 0.18^CDE^	9.56 ± 0.11^C^
UP-262	59.92 ± 1.12^ABC^	6.13 ± 0.08^DE^	19.53 ± 0.58^ABC^	63.32 ± 1.22^ABC^	20.47 ± 0.56^ABC^	72.57 ± 0.53^A^	92.08 ± 0.20^AB^	0.57 ± 0.04^A^	9.41 ± 0.05^CD^	92.56 ± 0.20^AB^	86.51 ± 0.25^H^	9.42 ± 0.05^CD^
WH-1105	58.82 ± 0.43^ABC^	6.06 ± 0.30^E^	18.65 ± 0.71^BCD^	62.01 ± 0.22^ABC^	19.61 ± 0.76^CD^	72.02 ± 0.31^AB^	92.54 ± 0.12^AB^	0.26 ± 0.03^FG^	8.52 ± 0.10^G^	92.93 ± 0.12^AB^	88.28 ± 0.20^AB^	8.52 ± 0.10^G^

*Data are presented as the mean ± SD. Means with different superscripts in column differ significantly (p ≤ 0.05). n = 3*.

The color in the wheat varieties is due to various pigments like carotenoids, flavonoids, anthocyanins, and some phenolic compounds (30), and the distribution of these compounds is affected by the genetic makeup of the cultivars, the geographical location, climate, and soil, which in turn results in color variations in wheat varieties (7). New breeding programs aimed at increasing the concentrations of these colored bioactive compounds in wheat and its products without affecting the yield can result in foods with superior bioactive and functional properties (7, 29).

### Flour Characteristics

#### Proximate Composition

The proximate composition of the different wheat varieties grown under different geographical conditions is given in Table 3. Moisture content varied from 8.67 to 12.65%. The protein content in the flour of the different wheat varieties varied from 9.32 to 12.60%. The protein content was found to be significantly (*p* ≤ 0.05) highest in the wheat variety HD-2967 and lowest in SW-1. Fat content was observed in the range between 0.91 and 1.51%. Ash content varied from 0.41 to 1.08%. Crude fiber ranged from 0.08 to 0.26%. The carbohydrate content, calculated after subtracting the values of moisture, fat, protein, ash, and fiber, ranged from 72.23 to 79.35% and was highest for SW-1 and lowest for HD-2967. Energy values ranged from 352.23 to 368.11 kcal/100 g. Significant differences (*p* ≤ 0.05) were observed in the moisture, fat, ash, protein, carbohydrate, and energy values among the cultivars. Fiber content showed a non-significant (*p* ≥ 0.05) difference among the cultivars. Considerable variations in the protein content of flour among the varieties revealed that these varieties can be used for varied wheat-based products. Previous studies have reported a moisture content of 7.79–9.35%, fat 2.62–3.48%, protein 9.03–12.33%, crude fiber 0.79–0.93%, and carbohydrate content of 72.6–76.5% (31). Memon et al. (32) obtained a moisture content of 7.13–7.61%, protein 10.9–11.8%, fat 0.12–0.25%, ash 2.10–2.77%, crude fiber 0.26–0.28%, carbohydrate 78.4–79.7%, and energy 358.99–363 kcal/100 g for flour of different wheat varieties grown in Pakistan. The chemical composition is dependent on the genetic makeup of the cultivars, climatic variations, irrigation practices, milling, soil fertility, and agricultural practices, which might explain the difference among various studies.

**Table 3:** Proximate composition, wet and dry gluten, and SDS volume of flours of the different wheat varieties of North-India.

**Variety**	**Moisture (%)**	**Protein (%)**	**Fat (%)**	**Ash (%)**	**Fiber (%)**	**Carbohydrate (%)**	**Energy (kcal/100 g)**	**Wet gluten (%)**	**Dry gluten (%)**	**SDS sedimentation volume (ml)**
DBW-17	10.12 ± 1.13^CDE^	12.10 ± 0.59^AB^	0.93 ± 0.03^EF^	1.08 ± 0.04^A^	0.15 ± 0.07^A^	75.62 ± 1.62^B^	359.25 ± 4.58^BCDE^	32.80 ± 0.80^EF^	12.23 ± 0.10^C^	40.50 ± 0.71^BCD^
HD-2851	11.01 ± 0.22^BCD^	10.71 ± 1.11^ABCD^	1.05 ± 0.16^CDEF^	0.48 ± 0.03^EF^	0.11 ± 0.01^A^	76.64 ± 1.28^AB^	358.83 ± 1.05^BCDE^	26.49 ± 0.73^G^	9.87 ± 0.12^D^	44.00 ± 1.41^ABC^
HD-2967	12.65 ± 0.10^A^	12.60 ± 0.66^A^	1.43 ± 0.10^AB^	0.89 ± 0.04^B^	0.20 ± 0.08^A^	72.23 ± 0.74^C^	352.23 ± 1.12^E^	38.09 ± 0.18^B^	13.34 ± 0.18^ABC^	49.00 ± 0.00^A^
HD-3086	10.18 ± 0.10^CDE^	11.24 ± 0.83^ABC^	1.22 ± 0.02^ABCDEF^	0.66 ± 0.03^D^	0.20 ± 0.02^A^	76.50 ± 0.91^AB^	361.90 ± 0.45^ABC^	37.38 ± 1.17^BC^	13.58 ± 0.38^ABC^	45.00 ± 0.00^AB^
PBW-175	10.54 ± 0.66^CD^	11.19 ± 0.70^ABCD^	0.93 ± 0.05^DEF^	0.71 ± 0.07^CD^	0.10 ± 0.05^A^	76.56 ± 1.35^AB^	359.40 ± 3.03^BCDE^	38.17 ± 0.68^B^	14.24 ± 0.18^AB^	34.50 ± 0.71^D^
PBW-502	10.23 ± 0.23^CDE^	11.81 ± 0.19^AB^	1.12 ± 0.15^BCDEF^	0.71 ± 0.03^CD^	0.15 ± 0.03^A^	75.98 ± 0.19^B^	361.26 ± 1.75^ABCD^	32.07 ± 1.24^EF^	12.06 ± 0.37^C^	41.50 ± 3.54^BCD^
PBW-550	12.15 ± 0.10^AB^	10.45 ± 0.25^BCD^	1.28 ± 0.10^ABCD^	0.63 ± 0.04^D^	0.25 ± 0.07^A^	75.25 ± 0.15^B^	354.28 ± 0.24^DE^	30.68 ± 0.68^F^	12.17 ± 0.38^C^	38.00 ± 1.41^BCD^
PBW-621	10.31 ± 0.09^CD^	10.98 ± 0.22^ABCD^	1.22 ± 0.19^ABCDEF^	0.81 ± 0.06^BC^	0.26 ± 0.09^A^	76.42 ± 0.13^B^	360.57 ± 1.02^BCD^	33.53 ± 0.38^DEF^	12.30 ± 2.01^C^	40.00 ± 2.83^BCD^
PBW-644	10.22 ± 0.15^CDE^	11.71 ± 0.38^ABC^	1.51 ± 0.04^A^	0.84 ± 0.02^B^	0.24 ± 0.12^A^	75.47 ± 0.30^B^	362.34 ± 0.42^ABC^	43.04 ± 1.90^A^	15.00 ± 0.23^A^	37.50 ± 0.70^CD^
PBW-660	10.45 ± 0.31^CD^	11.09 ± 0.59^ABCD^	1.38 ± 0.26^ABC^	0.60 ± 0.04^DE^	0.16 ± 0.04^A^	76.33 ± 0.71^B^	362.03 ± 2.32^ABC^	26.76 ± 0.150^G^	9.97 ± 0.15^D^	36.00 ± 1.41^D^
SW-1	9.61 ± 0.18^DE^	9.32 ± 0.55^D^	1.01 ± 0.04^DEF^	0.63 ± 0.08^D^	0.08 ± 0.08^A^	79.35 ± 0.54^A^	363.77 ± 0.49^AB^	27.56 ± 1.20^G^	9.63 ± 0.24^D^	39.50 ± 2.12^BCD^
SW-2	11.46 ± 0.12^ABC^	9.84 ± 0.81^CD^	0.91 ± 0.02^F^	0.70 ± 0.03^CD^	0.16 ± 0.07^A^	76.94 ± 0.89 ^AB^	355.29 ± 0.34^CDE^	23.46 ± 0.68^H^	8.28 ± 0.10^D^	44.00 ± 0.00^ABC^
UP-262	10.86 ± 1.46^BCD^	12.19 ± 0.49^AB^	1.01 ± 0.12^DEF^	0.41 ± 0.05^F^	0.21 ± 0.05^A^	75.32 ± 1.79^B^	359.19 ± 6.13^AB^	34.92 ± 1.30^CDE^	12.73 ± 0.26^BC^	37.50 ± 0.71^CD^
WH-1105	8.67 ± 0.12^E^	10.71 ± 1.11^ABCD^	1.27 ± 0.05^ABCDE^	0.67 ± 0.04^D^	0.21 ± 0.09^A^	77.84 ± 0.59^AB^	368.11 ± 1.21^A^	36.23 ± 1.39^BCD^	13.36 ± 0.10^ABC^	49.50 ± 3.54^A^

*Data are presented as the mean ± SD. Means with different superscripts in column differ significantly (p ≤ 0.05). n = 3*.

#### Dry and Wet Gluten Contents

The wet and dry gluten content of the different wheat varieties is given in Table 3. Wet gluten provides a quantitative measurement of the gluten-forming proteins in flour that are primarily responsible for the rheological properties and baking qualities of its dough (33). Wet gluten varied from 23.46 to 43.04%. The flour of the wheat variety PBW-644 recorded the significantly (*p* ≤ 0.05) highest wet gluten content and wheat variety SW-2 showed the lowest. The dry gluten content followed the same pattern as the wet gluten among the wheat varieties and varied from 8.28 to 15.00%. The variations in the gluten contents among the different wheat varieties arose due to the genetic makeup, cultivation practices, and fertilizer application (34), which support the significant difference found in the present study. The present results are closely related to the previous findings of Kundu et al. (8) who reported wet and dry gluten contents of different Indian wheat varieties between 14.49 and 43.70% and between 5.12 and 12.82%, respectively. However, lower values for wet and dry gluten contents (19.76–26.08 and 6.83–10.75%, respectively) have been reported by Asim et al. (35) for different Pakistani wheat varieties.

#### SDS Sedimentation Value

SDS sedimentation provides information about the protein quality of the wheat flour and is used to predict the gluten strength and baking quality (36). The SDS sedimentation value of flours from the different wheat cultivars ranged from 34.50 to 49.50 ml (Table 3). The flour of the wheat variety WH-1105 showed significantly the highest and PBW-175 the lowest SDS sedimentation value. The SDS sedimentation value has been reported to be due to the swelling of the glutenin strands (37), and dough with higher gluten strength results in higher swelling in the SDS solution, which results in higher sedimentation values. Wheat varieties having sedimentation values of <30 ml are more suitable for cookie making, 30–60 ml for chapatti/pasta making, and those with >60 ml for bread making (38). Kundu et al. (8) also observed that a sedimentation value of 35.7 ml resulted in good quality chapatti and that above 55 ml leads to poor quality chapatti. The results confirmed that the wheat varieties used in this study showed variable gluten strength, more suitable for making chapattis, and are of comparatively poor quality for bread and biscuit making. Similar results were obtained by Patil et al. (38), who reported that Indian wheat varieties were more suitable for making good to very good chapattis. SDS sedimentation values of 33–52 ml have also been reported by Panghal et al. (39) for different Indian wheat varieties, which are in concordance with our findings.

#### Flour Color

The CIE L^*^, a^*^, and b^*^ values of the flour from the different wheat varieties ranged from 90.82 to 92.88, 0.22 to 0.57, and from 7.71 to 10.80, respectively (Table 2). Significant (*p* ≤ 0.05) differences were observed in the color parameters (L^*^, a^*^, and b^*^) in the flour of the different wheat varieties. Flour of the wheat variety PBW-502 showed the highest and DBW-17 showed the lowest L^*^ value. The highest a^*^ value was reported for UP-262 flour, while HD-2851 flour showed the lowest value. Flour of the wheat variety SW-1 exhibited more yellowness in comparison to the other wheat varieties due to significantly (*p* ≤ 0.05) higher b^*^ values. On the contrary, flour of the wheat variety HD-3086 exhibited the lowest b^*^ value. The results are in conformity with Costa et al. (40), who obtained L^*^, a^*^, and b^*^ values of 92.94–95.42, 0.28–0.99, and 7.37–10.93 for flour of different Brazilian wheat genotypes. The color variations among flours of the different wheat varieties are mainly due to differences in the ash content, the contamination of flour with bran during milling, and, to some extent, the presence of naturally occurring pigments like anthocyanins, carotenoids, flavonoids, and some phenolic compounds (30), which are most concentrated in the aleurone layer and are removed during milling. The hue and chroma values varied significantly (*p* ≤ 0.05) from 86.51 to 88.54 and from 7.71 to 10.80, respectively. Wheat variety HD-2967 showed the highest and UP-262 the lowest hue value. The highest value for chroma was observed in SW-1 and DBW-17 and the lowest in HD-3086. Δ*E*, which is an indicator of total color difference, ranged from 91.32 to 93.29 for flours of the different wheat cultivars.

#### Solvent Retention Capacity

Solvent retention capacity (SRC) is the weight of the solvent retained by the wet, hydrated, and swollen flour pellets after centrifugation and is expressed as the percentage of flour weight. SRC is based on the principle of the swelling behavior of the different flour constituents in selected solvents: water (WSRC), lactic acid (LASRC), sodium carbonate (SCSRC), and sucrose (SUSRC) (41). SRC provides knowledge about the various chemical constituents of the flour during dough formation, its rheological properties, baking, and processing (21), which results in better finished product quality (22).

Table 4 lists the mean SRC values of the 14 Indian wheat varieties. LASRC mimics the acidic environment produced by lactic acid bacteria and characterizes the swelling of the glutenin fibrils of gluten proteins during fermentation, thus reflecting the dough strength. LASRC ranged from 80.45 to 113.70% and was found highest in the flour of wheat variety HD-3086 and lowest in the flour of UP-262, which reflected the high variability in gluten proteins among the wheat varieties. SCSRC, an indicator of starch damage and, indirectly, hardness, was highest for PBW-550 (97.95%) and lowest for WH-1105 (80.98%). SUSRC, associated with gliadin characteristics and the swelling of wheat flour arabinoxylans (22), ranged from 85.37 to 114.61%. PBW-550 showed the highest and UP-262 the lowest SUSRC. Water hydrates and swells up all three (gluten, starch, and pentosans) polymeric flour components (22). WSRC ranged from 69.70 to 87.53% and was found highest in UP-262 and lowest in SW-1. Duyvejonck et al. (41) found that wheat varieties with high WSRC and LASRC resulted in poor cookie making.

**Table 4:** Solvent retention capacity (SRC) of flours of the different wheat varieties of North-India.

**Variety**	**Water (WSRC)**	**Sucrose (SUSRC)**	**Lactic acid (LASRC)**	**Sodium carbonate (SCSRC)**	**Gluten performance index (GPI)**
DBW-17	74.72 ± 0.14^DEFG^	107.78 ± 3.18^ABC^	90.08 ± 0.20^F^	86.68 ± 1.22^CDE^	0.46 ± 0.01^F^
HD-2851	75.62 ± 0.75^DEFG^	101.33 ± 3.62^CD^	93.74 ± 1.64^EF^	87.36 ± 2.32^CDE^	0.50 ± 0.01^CDEF^
HD-2967	78.20 ± 0.28^BCDE^	103.22 ± 3.05^BCD^	105.92 ± 2.84^BC^	81.88 ± 0.35^E^	0.57 ± 0.02^A^
HD-3086	78.23 ± 0.95^BCDE^	103.36 ± 3.45^BCD^	113.70 ± 1.15^A^	89.67 ± 0.34^BCD^	0.59 ± 0.01^A^
PBW-175	82.65 ± 0.54^ABC^	111.80 ± 0.00^AB^	94.95 ± 0.27^EF^	91.25 ± 0.07^BCD^	0.47 ± 0.00^EF^
PBW-502	80.28 ± 3.25^BCD^	101.84 ± 3.25^CD^	94.89 ± 1.83^EF^	91.87 ± 2.44^ABCD^	0.49 ± 0.00^DEF^
PBW-550	84.14 ± 1.53^AB^	114.61 ± 3.75^A^	110.88 ± 1.67^AB^	97.95 ± 0.90^A^	0.52 ± 0.00^BCD^
PBW-621	70.76 ± 0.54^FG^	100.25 ± 2.64^CDE^	102.60 ± 0.54^CD^	81.93 ± 1.29^E^	0.56 ± 0.01^AB^
PBW-644	74.28 ± 2.91^DEFG^	102.11 ± 1.90^BCD^	90.19 ± 2.91^F^	85.21 ± 1.02^DE^	0.48 ± 0.01^DEF^
PBW-660	76.59 ± 2.92^CDEF^	100.55 ± 1.55^CDE^	93.78 ± 2.24^EF^	88.64 ± 0.82^BCD^	0.50 ± 0.01^DEF^
SW-1	87.53 ± 2.56^A^	98.71 ± 1.55^CDE^	99.33 ± 2.83^CDE^	95.29 ± 0.34^AB^	0.51 ± 0.02^CDE^
SW-2	75.23 ± 1.72^DEFG^	90.87 ± 0.48^EF^	90.14 ± 0.69^F^	93.29 ± 2.68^ABC^	0.49 ± 0.00^DEF^
UP-262	69.70 ± 0.48^G^	85.37 ± 1.64^F^	80.45 ± 2.59^G^	81.22 ± 3.82^E^	0.48 ± 0.02^DEF^
WH-1105	72.13 ± 0.13^EFG^	95.44 ± 1.00^DE^	95.86 ± 0.27^DEF^	80.98 ± 0.27^E^	0.54 ± 0.00^ABC^

*Data are presented as the mean ± SD. Means with different superscripts in column differ significantly (p ≤ 0.05). n = 3*.

The WSRC, LASRC, SCSRC, and SUSRC values were reported in ranges of 49.4–56.3, 90–118.5, 66–83.0, and 86.4–106.3%, respectively, for US soft wheat cultivars (42). Ram et al. (21) reported WSRC values of 53.4–70.6%, LASRC of 72.0–122.8%, SCSRC of 63.9–87.2%, and SUSRC of 75.1–97.9% across 192 Indian wheat varieties. So our results are within the range reported by these authors. However, higher SRC values have been reported by Lindgren and Simsek (43) for hard red spring wheat cultivars: WSRC of 69.6–104.9%, LASRC of 148.5–178.1%, SCSRC of 86.0–160.2%, and SUSRC of 128.2–157.5%. Similarly, high SRC values for different Pakistani wheat varieties have been reported by Pasha et al. (44) owing to their semi-soft to medium-hard nature. Higher SRC values are usually associated with better baking qualities (28). The low LASRC values indicated that the wheat varieties in the current study are in the soft to the semi-soft range, having medium-strong gluten, and are more suitable for making chapattis and biscuits. A similar observation regarding Indian wheat varieties has been made by Ram et al. (21). Karaduman (45) also reported LASRC as an effective, fast, and reliable method for differentiating between soft and hard wheat varieties. Hammed et al. (46) also reported higher SRC values for hard wheat varieties owing to their high protein content, greater gluten strength, greater starch damage, high arabinoxylan content, and high water absorption capacity.

Gluten performance index (GPI) provides the most reliable information about gluten strength, functionality, and baking performance and is considered a better predictor to determine the overall performance of flour glutenin (22). GPI ranged from 0.46 to 0.59 (Table 5) and varied significantly (*p* ≤ 0.05) among flours of the different wheat cultivars. HD-3086 showed the highest GPI value of 0.46 and DBW-17 the lowest GPI value of 0.59. Jeon et al. (42) reported GPI values of 0.52–0.69 for soft wheat cultivars. Our results are in close range with those reported. However, higher values have been reported by Lindgren and Simsek (43) (0.47–0.80) and Hammed et al. (46) (0.62–0.85) for hard wheat varieties. GPI is directly related to LASRC and can be detrimentally minimized with higher values of SUSRC and SCSRC. In our study, the values of LASRC, SUSRC, and SCSRC are almost the same, which might explain the observed values of GPI and also the type of wheat varieties used in this study.

**Table 5:** Non-essential amino acid (NEAA) content in flours of the different wheat varieties of North-India (in grams per 100 g protein).

**Variety**	**Aspartic acid**	**Glutamic acid**	**Serine**	**Glycine**	**Alanine**	**Arginine**	**Tyrosine**	**Cystine**	**Proline**	**Total NEAA**
DBW-17	6.76 ± 0.32^A^	31.99 ± 1.15^E^	4.23 ± 0.29^A^	4.31 ± 0.06^H^	5.81 ± 0.66^BC^	2.27 ± 0.07^A^	3.20 ± 0.02^A^	4.32 ± 0.35^A^	6.39 ± 0.55^DEF^	69.28 ± 2.23^BC^
HD-2851	3.63 ± 0.35^CDE^	32.27 ± 0.26^DE^	4.48 ± 0.50^A^	4.53 ± 0.25^H^	6.44 ± 0.40^BC^	2.10 ± 0.29^A^	3.03 ± 0.41^A^	0.68 ± 0.07^B^	14.98 ± 0.44^A^	72.12 ± 1.95^ABC^
HD-2967	4.11 ± 0.32^BCDE^	33.90 ± 0.12^CD^	5.26 ± 0.63^A^	4.64 ± 0.20^GH^	7.26 ± 0.88^ABC^	2.75 ± 0.58^A^	3.72 ± 0.70^A^	3.60 ± 2.86^AB^	3.93 ± 1.37^F^	69.17 ± 0.80^BC^
HD-3086	4.00 ± 0.02^CDE^	34.96 ± 0.11^BC^	5.11 ± 0.29^A^	6.25 ± 0.07^ABCD^	7.03 ± 0.16^ABC^	2.71 ± 0.36^A^	3.71 ± 0.54^A^	1.53 ± 0.71^AB^	5.99 ± 1.22^EF^	71.29 ± 0.75^ABC^
PBW-175	4.90 ± 0.16^B^	36.84 ± 0.27^AB^	4.96 ± 0.26^A^	5.51 ± 0.39^DEFG^	7.19 ± 0.91^ABC^	2.65 ± 0.10^A^	3.90 ± 0.11^A^	1.29 ± 0.12^AB^	5.27 ± 0.56^EF^	72.50 ± 0.76^AB^
PBW-502	4.10 ± 0.01^BCDE^	34.40 ± 0.45^C^	4.17 ± 0.46^A^	4.87 ± 0.16^FGH^	6.35 ± 0.38^BC^	2.54 ± 0.34^A^	3.84 ± 0.51^A^	0.73 ± 0.08^B^	9.05 ± 0.60^BCD^	70.05 ± 1.46^ABC^
PBW-550	4.10 ± 0.19^BCDE^	37.18 ± 0.85^A^	4.81 ± 0.53^A^	6.60 ± 0.52^AB^	7.96 ± 0.48^AB^	2.85 ± 0.05^A^	3.87 ± 0.30^A^	0.99 ± 0.62^B^	7.81 ± 0.88^CDE^	76.19 ± 0.11^A^
PBW-621	3.28 ± 0.42^E^	34.06 ± 0.08^CD^	4.76 ± 0.69^A^	5.17 ± 0.27^EFGH^	6.85 ± 0.65^ABC^	2.28 ± 0.39^A^	3.13 ± 0.52^A^	0.77 ± 0.11^B^	7.91 ± 0.25^CDE^	68.19 ± 2.88^BC^
PBW-644	4.40 ± 0.23^BCD^	32.35 ± 0.45^DE^	4.56 ± 0.08^A^	6.00 ± 0.14^BCDE^	6.15 ± 0.48^BC^	2.34 ± 0.03^A^	3.43 ± 0.26^A^	0.86 ± 0.11^B^	11.36 ± 0.43^B^	71.44 ± 0.87^ABC^
PBW-660	4.51 ± 0.07^BC^	34.62 ± 0.36^CD^	4.37 ± 0.18^A^	5.55 ± 0.07^CDEF^	8.70 ± 0.28^A^	3.35 ± 0.03^A^	3.99 ± 0.18^A^	1.62 ± 0.08^AB^	5.61 ± 0.38^EF^	72.30 ± 0.03^ABC^
SW-1	3.72 ± 0.07^CDE^	30.83 ± 0.71^E^	4.75 ± 0.05^A^	5.98 ± 0.11^BCDE^	6.72 ± 0.22^ABC^	2.59 ± 0.27^A^	3.19 ± 0.13^A^	4.29 ± 0.09^A^	7.94 ± 0.39^CDE^	70.00 ± 0.79^ABC^
SW-2	3.33 ± 0.10^E^	31.13 ± 0.18^E^	4.90 ± 0.15^A^	7.11 ± 0.13^A^	5.62 ± 0.44^C^	3.06 ± 0.47^A^	4.10 ± 0.62^A^	0.83 ± 0.10^B^	5.78 ± 0.27^EF^	65.85 ± 1.93^C^
UP-262	3.45 ± 0.18^E^	30.53 ± 0.69^E^	4.40 ± 0.43^A^	6.43 ± 0.11^ABC^	5.88 ± 0.50^BC^	2.31 ± 0.37^A^	3.16 ± 0.50^A^	0.76 ± 0.38^B^	11.94 ± 1.27^B^	68.87 ± 3.28^BC^
WH-1105	3.55 ± 0.15^DE^	31.10 ± 0.22^E^	5.18 ± 0.12^A^	6.78 ± 0.14^AB^	6.72 ± 0.58^ABC^	2.64 ± 0.43^A^	3.13 ± 0.50^A^	0.74 ± 0.10^B^	9.65 ± 0.39^BC^	69.50 ± 1.15^BC^

*Data are presented as the mean ± SD. Means with different superscripts in column differ significantly (p ≤ 0.05) between varieties and means with similar superscripts differ non-significantly (p ≥ 0.05). n = 3 for each treatment*.

### Amino Acid Composition of Wheat Varieties

Figures S1A,B display the HPLC chromatogram of a standard mixture of 18 amino acids and flour of one of the wheat varieties, respectively. All the amino acids were separated within a run time of <40 min, having well-separated peaks. The retention time of the samples was comparable to that of the standard mixture.

The amino acid composition of the wheat varieties from different regions of North India is summarized in Tables 5, 6. The results indicated a considerable difference in the amount of amino acid among the wheat cultivars. Glutamic acid was found to be the most abundant amino acid, with mean concentrations of 30.53–37.18 g/100 g protein (Table 5). The highest concentration of glutamic acid was observed in the wheat variety PBW-550 and was lowest in UP-262. The results are inconsistent with those of Wang et al. (47), who found glutamic acid as the most dominant amino acid in the vicinity of 30.99–31.41 g/100 g protein in a Chinese wheat variety. Jood et al. (48) also found glutamic acid as the predominant amino acid, having a concentration of 31 g/100 g protein in an Indian wheat variety. Anjum et al. (15), however, reported glutamic acid contents of 6.29–12.03 g/100 g protein in different Pakistani wheat varieties, which are relatively lower than those observed in this study and numerous other studies.

**Table 6:** Essential amino acid (EAA) contents in flours of the different wheat varieties of North-India (in grams/100g protein).

**Name**	**Threonine**	**Histidine**	**Valine**	**Methionine**	**Phenylalanine**	**Isoleucine**	**Leucine**	**Lysine**	**Total EAA**
DBW-17	1.55 ± 0.09^ABC^	1.40 ± 0.07^ABC^	2.28 ± 0.13^ABCD^	1.35 ± 0.17^BC^	5.05 ± 0.10^AB^	3.30 ± 0.09^A^	7.11 ± 0.66^AB^	1.62 ± 0.04^EF^	23.67 ± 0.95^A^
HD-2851	0.94 ± 0.18^C^	0.87 ± 0.18^C^	1.32 ± 0.10^CDE^	1.84 ± 0.21^ABC^	3.79 ± 0.27^AB^	3.04 ± 0.21^A^	8.32 ± 0.06^AB^	3.61 ± 0.37^A^	23.71 ± 0.80^A^
HD-2967	1.54 ± 0.07 ^ABC^	1.33 ± 0.24^ABC^	1.65 ± 1.03^BCDE^	1.58 ± 0.52^ABC^	4.39 ± 1.68^AB^	3.27 ± 0.08^A^	8.68 ± 1.08^AB^	1.27 ± 0.09^F^	23.71 ± 1.42^A^
HD-3086	1.48 ± 0.04 ^ABC^	1.59 ± 0.14^ABC^	2.78 ± 0.18^AB^	1.52 ± 0.05^BC^	4.96 ± 1.10^AB^	3.37 ± 0.35^A^	8.79 ± 0.37^AB^	2.02 ± 0.02^BCDEF^	26.52 ± 1.10^A^
PBW-175	1.96 ± 0.03^A^	1.86 ± 0.17^A^	2.54 ± 0.44^ABCD^	1.19 ± 0.37^BC^	5.56 ± 0.16^A^	3.77 ± 0.30^A^	8.68 ± 0.49^AB^	1.57 ± 0.18^EF^	27.14 ± 0.09^A^
PBW-502	1.28 ± 0.24^BC^	1.58 ± 0.32^ABC^	2.07 ± 0.08^BCDE^	0.82 ± 0.43^BC^	3.45 ± 0.33^AB^	2.98 ± 0.20^A^	8.31 ± 0.57^AB^	3.38 ± 0.67^AB^	23.86 ± 1.50^A^
PBW-550	1.00 ± 0.01^C^	1.33 ± 0.33^ABC^	2.68 ± 0.20^ABC^	0.57 ± 0.25^C^	3.87 ± 0.16^AB^	3.32 ± 0.51^A^	9.06 ± 0.37^AB^	1.98 ± 0.29^CDEF^	23.80 ± 0.13^A^
PBW-621	1.51 ± 0.24 ^ABC^	1.18 ± 0.08^ABC^	3.52 ± 0.39^A^	1.12 ± 0.04^BC^	3.36 ± 0.14^AB^	2.85 ± 0.29^A^	7.87 ± 0.77^AB^	2.72 ± 0.88^ABCDE^	24.12 ± 1.92^A^
PBW-644	1.41 ± 0.25 ^ABC^	1.04 ± 0.09^BC^	2.25 ± 0.28^ABCD^	1.61 ± 0.21^ABC^	4.47 ± 0.27^AB^	2.80 ± 0.24^A^	6.99 ± 1.26^AB^	3.33 ± 0.11^ABC^	23.91 ± 0.53^A^
PBW-660	1.18 ± 0.11^BC^	1.00 ± 0.02^BC^	1.23 ± 0.18^DE^	1.03 ± 0.11^BC^	2.83 ± 0.10^B^	2.74 ± 0.08^A^	6.31 ± 0.21^B^	2.84 ± 0.20^ABCDE^	19.13 ± 0.54^B^
SW-1	1.64 ± 0.200^AB^	1.78 ± 0.29^AB^	2.38 ± 0.03^ABCD^	2.14 ± 0.35^AB^	5.32 ± 0.54^A^	3.22 ± 0.10^A^	7.31 ± 0.71^AB^	1.87 ± 0.16^DEF^	25.66 ± 0.95^A^
SW-2	1.40 ± 0.04 ^ABC^	1.24 ± 0.15^ABC^	1.90 ± 0.11^BCDE^	1.97 ± 0.19^AB^	4.75 ± 0.28^AB^	3.65 ± 0.32^A^	9.30 ± 0.43^A^	3.22 ± 0.08^ABCD^	27.43 ± 0.02^A^
UP-262	1.40 ± 0.24 ^ABC^	1.08 ± 0.25^ABC^	0.82 ± 0.11^E^	2.88 ± 0.14^A^	4.57 ± 0.24^AB^	3.06 ± 0.29^A^	7.97 ± 0.29^AB^	2.87 ± 0.12^ABCDE^	24.62 ± 0.43^A^
WH-1105	1.55 ± 0.07 ^ABC^	0.82 ± 0.19^C^	2.57 ± 0.14^ABCD^	1.59 ± 0.80^ABC^	4.43 ± 0.23^AB^	2.98 ± 0.28^A^	7.37 ± 1.38^AB^	3.37 ± 0.18^AB^	24.70 ± 0.93^A^

*Data are presented as the mean ± SD. Means with different superscripts in column differ significantly (p ≤ 0.05). n = 3*.

The amino acid concentrations (in grams per 100 g of protein) in flours of the different wheat varieties varied: aspartic, 3.28–6.76; serine, 4.17–5.18; glycine, 4.31–7.11; alanine, 5.62–8.70; arginine, 2.10–3.35; tyrosine, 3.03–4.10; cystine, 0.68–4.32; proline, 3.32–14.98; threonine, 0.94–1.96; histidine, 0.82–1.86; valine, 0.82–3.52; methionine, 0.57–2.88; phenylalanine, 2.83–5.56; isoleucine, 2.74–3.77; leucine, 6.31–9.30; and lysine, 1.27–3.61 (Tables 5, 6). Tryptophan got destroyed and was not detected. Significant (*p* ≤ 0.05) differences were observed in all essential amino acids. Among the non-essential amino acids, aspartic acid, glycine, alanine, arginine, cystine, and proline showed significant (*p* ≤ 0.05) differences, whereas serine, tyrosine, and arginine varied non-significantly (*p* ≥ 0.05). Anjum et al. (15); Wang et al. (47); Jood et al. (48) and Alijosius et al. (49) reported the following concentrations of amino acids (in grams per 100 g protein): aspartic acid, 3.93–6.60; serine, 3.35–5.69; glycine, 2.81–6.78; alanine, 0.30–4.02; arginine, 1.55–7.01; tyrosine, 1.39–4.82; cystine, 2.07–2.30; proline, 7.17–15.38; threonine, 2.40–4.05; histidine, 1.23–5.24; valine, 2.99–6.59; methionine, 0.75–2.70; phenylalanine, 4.21–5.47; isoleucine, 1.89–4.04; leucine, 3.78–7.62; and lysine, 2.05–3.14. Most of the values obtained agreed satisfactorily and were within the range; however, the mean concentration of alanine was somewhat higher and those of threonine, histidine, and valine were lower than those reported previously. Differences in the amino acid composition in flours of the different wheat varieties are attributed to growing environmental conditions like CO_2_ concentration and temperature (47), wheat types hard, soft, or medium, the protein content of the flour, the extraction rate (50), the genetic makeup of the cultivars, and the application of fertilizers (15, 51).

The non-essential amino acids (NEAA) comprising aspartic acid, glutamic acid, serine, glycine, alanine, arginine, tyrosine, cysteine, and proline constituted 65.85–76.19% of the total amino acids (Table 5). The NEAA are associated with gluten proteins (gliadin + glutenin) and play an important role in the end product use of wheat flour. The essential amino acids (EAA), which included threonine, histidine, valine, methionine, phenylalanine, isoleucine, leucine, and lysine (Table 6), accounted for 19.13–27.43% of the total amino acids. Statistically, significant (*p* ≤ 0.05) differences were observed in both NEAA and EAA. The flour of wheat variety PBW-550 was observed as having the highest and that of SW-2 the lowest percentage of NEAA. The highest proportion of EAA was observed in the wheat variety SW-2 and the lowest in PBW-660. The amino acid score (AAS) is used to predict the completeness of a particular protein. A score of 100% or more represents the EAA in the particular protein being equal to or more than the reference protein, and the protein is termed as a complete protein; however, if the score is <100, the protein under study is termed as an incomplete protein. The individual and total AAS of EAA are given in Table 7. Most of the scores were <100%, which indicated that the test protein was incomplete. The mean AAS of threonine, valine, phenylalanine, histidine, methionine, and lysine in almost all wheat varieties was <100%. Leucine and isoleucine had AAS of either more than 100% or close to 100% in most wheat varieties. The AAS of lysine was the lowest, having a mean score of 44.70%, and was thus the first limiting amino acid in the flour of the different wheat varieties. Jiang et al. (52) also reported lysine as the first limiting amino acid in different wheat varieties, with a mean AAS of 49.8%. Similar results have also been reported by Anjum et al. (15) and Jood et al. (48). The total amino acid score ranged from 58.33 to 83.64%. Significant (*p* ≤ 0.05) differences were observed in the total amino acid score between the wheat varieties. Wheat variety SW-2 showed the highest and PBW-660 the lowest total amino acid score. The higher EEA as well as the total amino acid score for wheat variety SW-2 may be attributed to the low amount of gluten proteins and higher albumin + globulin content (data not shown, article in press).

**Table 7:** Individual and total amino acid score (AAS) in flours of the different wheat varieties of North-India.

**Variety**	**Threonine**	**Histidine**	**Valine**	**Methionine**	**Phenylalanine**	**Isoleucine**	**Leucine**	**Lysine**	**Total AAS**
DBW-17	50.14 ± 2.99^ABC^	70.17 ± 3.29^ABC^	53.08 ± 3.05^ABCD^	49.98 ± 6.25^BC^	97.18 ± 2.00^AB^	103.00 ± 2.92^A^	107.79 ± 9.95^AB^	28.40 ± 0.78^EF^	72.17 ± 2.90^A^
HD-2851	30.19 ± 5.76^C^	43.31 ± 8.97^C^	30.61 ± 2.42^CDE^	67.97 ± 7.58^ABC^	72.90 ± 5.14^AB^	94.93 ± 6.68^A^	126.00 ± 0.95^AB^	63.27 ± 6.55^A^	72.27 ± 2.43^A^
HD-2967	49.59 ± 2.16^ABC^	66.68 ± 11.97^ABC^	38.5 ± 23.9B^CDE^	58.40 ± 19.30^ABC^	84.30 ± 32.30^AB^	102.30 ± 2.57^A^	131.50 ± 16.30^AB^	22.29 ± 1.50^F^	72.28 ± 4.33^A^
HD-3086	47.68 ± 1.27^ABC^	79.38 ± 6.79^ABC^	64.74 ± 4.24^AB^	56.30 ± 1.81^BC^	95.40 ± 21.1^AB^	105.32 ± 11.01^A^	133.19 ± 5.64^AB^	35.48 ± 0.41^BCDEF^	80.84 ± 3.34^A^
PBW-175	63.23 ± 0.91^A^	93.15 ± 8.27^A^	59.08 ± 10.21^ABCD^	44.09 ± 13.60^BC^	106.93 ± 2.98^A^	117.83 ± 9.25^A^	131.56 ± 7.36^AB^	27.61 ± 3.07^EF^	82.75 ± 0.27^A^
PBW-502	41.33 ± 7.98^BC^	78.70 ± 16.10^ABC^	48.03 ± 1.82^BCDE^	30.30 ± 15.80^BC^	66.34 ± 6.25^AB^	93.23 ± 6.35^A^	125.94 ± 8.61^AB^	59.24 ± 11.82^AB^	72.75 ± 4.56^A^
PBW-550	32.39 ± 0.42^C^	66.30 ± 16.60^ABC^	62.42 ± 4.68^ABC^	20.97 ± 9.27^C^	74.35 ± 3.09^AB^	103.70 ± 15.80^A^	137.20 ± 5.60^AB^	34.75 ± 5.02^CDEF^	72.56 ± 0.39^A^
PBW-621	48.60 ± 7.70^ABC^	58.81 ± 3.80^ABC^	81.76 ± 9.07^A^	41.37 ± 1.42^BC^	64.67 ± 2.77^AB^	89.09 ± 9.20^A^	119.19 ± 11.71^AB^	47.70 ± 15.40^ABCDE^	73.53 ± 5.84^A^
PBW-644	45.35 ± 7.91^ABC^	51.91 ± 4.38^BC^	52.34 ± 6.56^ABCD^	59.70 ± 7.76^ABC^	86.04 ± 5.09^AB^	87.64 ± 7.62^A^	106.00 ± 19.00^AB^	58.46 ± 2.04^ABC^	72.90 ± 1.61^A^
PBW-660	37.90 ± 3.42^BC^	49.75 ± 1.06^BC^	28.49 ± 4.11^DE^	37.96 ± 3.93^BC^	54.42 ± 1.90^B^	85.53 ± 2.35^A^	95.53 ± 3.11^B^	49.82 ± 3.47^ABCDE^	58.33 ± 1.63^B^
SW-1	52.88 ± 6.41^AB^	88.80 ± 14.40^AB^	55.25 ± 0.804^ABCD^	79.41 ± 12.88^AB^	102.32 ± 10.31^A^	100.65 ± 3.05^A^	110.75 ± 10.70^AB^	32.79 ± 2.75^DEF^	78.22 ± 2.91^A^
SW-2	45.30 ± 1.17^ABC^	62.22 ± 7.47^ABC^	44.27 ± 2.51^BCDE^	72.80 ± 7.05^AB^	91.25 ± 5.36^AB^	113.96 ± 9.96^A^	140.97 ± 6.52^A^	56.50 ± 1.47^ABCD^	83.64 ± 0.08^A^
UP-262	45.15 ± 7.60^ABC^	53.81 ± 12.34^ABC^	18.95 ± 2.48^E^	106.61 ± 5.31^A^	87.83 ± 4.65^AB^	95.57 ± 8.90^A^	120.67 ± 4.38^AB^	50.30 ± 2.16^ABCDE^	75.08 ± 1.31^A^
WH-1105	50.06 ± 2.20^ABC^	40.80 ± 9.40^C^	59.85 ± 3.17^ABCD^	59.00 ± 29.50^ABC^	85.27 ± 4.43^AB^	93.21 ± 8.78^A^	111.70 ± 20.90^AB^	59.19 ± 3.07^AB^	75.30 ± 2.82^A^
**Mean**	**45.70** **±** **9.02**	**64.56** **±** **17.48**	**49.81** **±** **17.27**	**56.06** **±** **23.20**	**83.52** **±** **17.02**	**99.00** **±** **11.08**	**121.28** **±** **15.30**	**44.70** **±** **14.39**	**74.47** **±** **6.41**
FAO^*^	**3.1**	**2**	**4.3**	**2.7**	**5.2**	**3.2**	**6.6**	**5.7**	

*Data are presented as the mean ± SD. Means with different superscripts in column differ significantly (p ≤ 0.05). n = 3. The bold values are mean values representing total amino acid score (AAS) of different essential amino acids in different wheat varieties*.

* *Amino Acid Score based on FAO (2013) pattern for a child (6 months to 3 years)*.

The study was further extended to determine the proportions of the different amino acid groups (Table 8). The total percentage of amino acids was in the range of 91.43–99.99 g/100 g protein for flours of the different wheat varieties. Basic amino acids constituted 5.29–7.52 g/100 g protein of the total amino acids and were statistically non-significant (*p* ≥ 0.05). Acidic amino acids accounted for 33.98–41.74 g/100 g protein of the total amino acids and were significantly (*p* ≤ 0.05) highest for PBW-175 and lowest for UP-262. The results demonstrated wheat flour to be more acidic in nature. Aromatic amino acids constituted 6.49–9.46 g/100 g protein of the total amino acids and showed a significant (*p* ≤ 0.05) difference, being highest for PBW-175 and lowest for PBW-621. The proportion of non-polar amino acids accounted for 29.43–38.61 g/100 g protein of the total amino acids, being significantly (*p* ≤ 0.05) highest for HD-2851 and lowest for UP-262. Polar and sulfur amino acids accounted for 7.00–12.82 g/100 g protein and 1.55–6.43 g/100 g protein of the total amino acids, respectively. Statistically, the highest percentages of both polar and sulfur amino acids were observed in SW-1 and the lowest in PBW-502. Hydroxy amino acids accounted for 5.42–6.92 g/100 g protein of the total amino acids. The highest proportion of sulfur amino acids was observed in PBW-175 and the lowest in HD-2851. The arginine/lysine ratio varied significantly (*p* ≤ 0.05) from 0.59 to 2.19, being highest for HD-2851 and lowest for HD-2967.

**Table 8:** Total amino acids (AA) and proportions of the different amino acid groups in flours of the different wheat varieties of North-India.

**Name**	**Total AA**	**Basic amino acids**	**Acidic amino acids**	**Aromatic amino acids**	**Non-polar AA**	**Polar AA**	**Hydroxy AA**	**Sulfur AA**	**Arginine/Lysine**
DBW-17	92.95 ± 3.18^A^	5.29 ± 0.18^A^	38.76 ± 1.47^CD^	8.25 ± 0.13^ABCD^	29.20 ± 1.05^D^	11.45 ± 0.72^ABC^	5.78 ± 0.20^ABC^	5.67 ± 0.52^AB^	1.40 ± 0.00^BC^
HD-2851	95.83 ± 1.15^A^	6.57 ± 0.09^A^	35.89 ± 0.09^EFG^	6.82 ± 0.14^CD^	38.61 ± 0.64^A^	7.93 ± 0.19^DE^	5.42 ± 0.32^C^	2.52 ± 0.13^CD^	0.59 ± 0.14^D^
HD-2967	92.88 ± 2.22^A^	5.36 ± 0.26^A^	38.01 ± 0.44^CDE^	8.10 ± 0.99^ABCD^	29.43 ± 0.16^D^	11.98 ± 1.78^AB^	6.80 ± 0.56^A^	5.18 ± 2.34^ABC^	2.19 ± 0.61^A^
HD-3086	97.81 ± 1.84^A^	6.32 ± 0.25^A^	38.96 ± 0.09^CD^	8.68 ± 0.56^AB^	34.22 ± 0.93^ABCD^	9.64 ± 0.51^BCD^	6.59 ± 0.25^ABC^	3.05 ± 0.76^BCD^	1.34 ± 0.16^BCD^
PBW-175	99.64 ± 0.85^A^	6.08 ± 0.11^A^	41.74 ± 0.43^A^	9.46 ± 0.27^A^	32.96 ± 0.59^BCD^	9.40 ± 0.02^CDE^	6.92 ± 0.23^A^	2.48 ± 0.25^CD^	1.69 ± 0.13^AB^
PBW-502	93.91 ± 2.95^A^	7.49 ± 1.34^A^	38.50 ± 0.46^CD^	7.29 ± 0.83^BCD^	33.63 ± 0.47^ABCD^	7.00 ± 0.14^E^	5.47 ± 0.21^C^	1.55 ± 0.35^D^	0.76 ± 0.05^CD^
PBW-550	99.99 ± 0.02^A^	6.16 ± 0.67^A^	41.28 ± 0.47^AB^	7.74 ± 0.13^ABCD^	37.43 ± 0.90^AB^	7.38 ± 0.36^DE^	5.82 ± 0.51^ABC^	1.56 ± 0.87^D^	1.45 ± 0.18^ABC^
PBW-621	92.31 ± 4.79^A^	6.18 ± 1.19^A^	37.33 ± 0.50^CDE^	6.49 ± 0.38^D^	34.16 ± 2.13^ABCD^	8.15 ± 0.60^DE^	6.26 ± 0.45^ABC^	1.89 ± 0.15^D^	0.86 ± 0.14^CD^
PBW-644	95.35 ± 1.39^A^	6.71 ± 0.06^A^	36.75 ± 0.67^DEF^	7.91 ± 0.01^ABCD^	35.55 ± 2.27^ABC^	8.43 ± 0.27^DE^	5.96 ± 0.16^ABC^	2.47 ± 0.10^CD^	0.70 ± 0.01^CD^
PBW-660	91.43 ± 0.57^A^	7.19 ± 0.21^A^	39.13 ± 0.29^BC^	6.82 ± 0.28^CD^	30.12 ± 1.19^CD^	8.19 ± 0.26^DE^	5.55 ± 0.29^BC^	2.64 ± 0.03^BCD^	1.18 ± 0.07^BCD^
SW-1	95.66 ± 0.17^A^	6.23 ± 0.72^A^	34.55 ± 0.78^FG^	8.51 ± 0.41^ABC^	33.55 ± 0.86^ABCD^	12.82 ± 0.68^A^	6.39 ± 0.25^ABC^	6.43 ± 0.44^A^	1.38 ± 0.03^BC^
SW-2	93.28 ± 1.90^A^	7.52 ± 0.29^A^	34.46 ± 0.28^FG^	8.83 ± 0.34^AB^	33.36 ± 0.95^ABCD^	9.11 ± 0.10^CDE^	6.31 ± 0.18^ABC^	2.80 ± 0.09^BCD^	0.95 ± 0.17^BCD^
UP-262	93.49 ± 3.71^A^	6.26 ± 0.74^A^	33.98 ± 0.50^G^	7.73 ± 0.26^ABCD^	36.09 ± 2.55^AB^	9.44 ± 0.33B^CDE^	5.80 ± 0.19^ABC^	3.63 ± 0.52^ABCD^	0.80 ± 0.09^CD^
WH-1105	94.20 ± 2.08^A^	6.83 ± 0.79^A^	34.65 ± 0.07^FG^	7.57 ± 0.27^BCD^	36.08 ± 1.85^AB^	9.07 ± 0.75^CDE^	6.74 ± 0.05^AB^	2.33 ± 0.70^CD^	0.78 ± 0.09^CD^

*Data are presented as the mean ± SD. Means with different superscripts in column differ significantly (p ≤ 0.05). n = 3*.

*Basic amino acids: arginine + lysine + histidine. Acidic amino acids: aspartic + glutamic acid. Aromatic amino acids: phenylalanine + tryptophan + tyrosine. Non-polar amino acids: glycine, alanine + valine + isoleucine + leucine + proline. Polar amino acids: cysteine + methionine + serine + threonine. Hydroxy amino acids: serine + threonine. Sulfur amino acids: cysteine + methionine*.

*Arg/Lys, arginine/lysine*.

### SDS-PAGE of Wheat Flour

**Figure 1:**
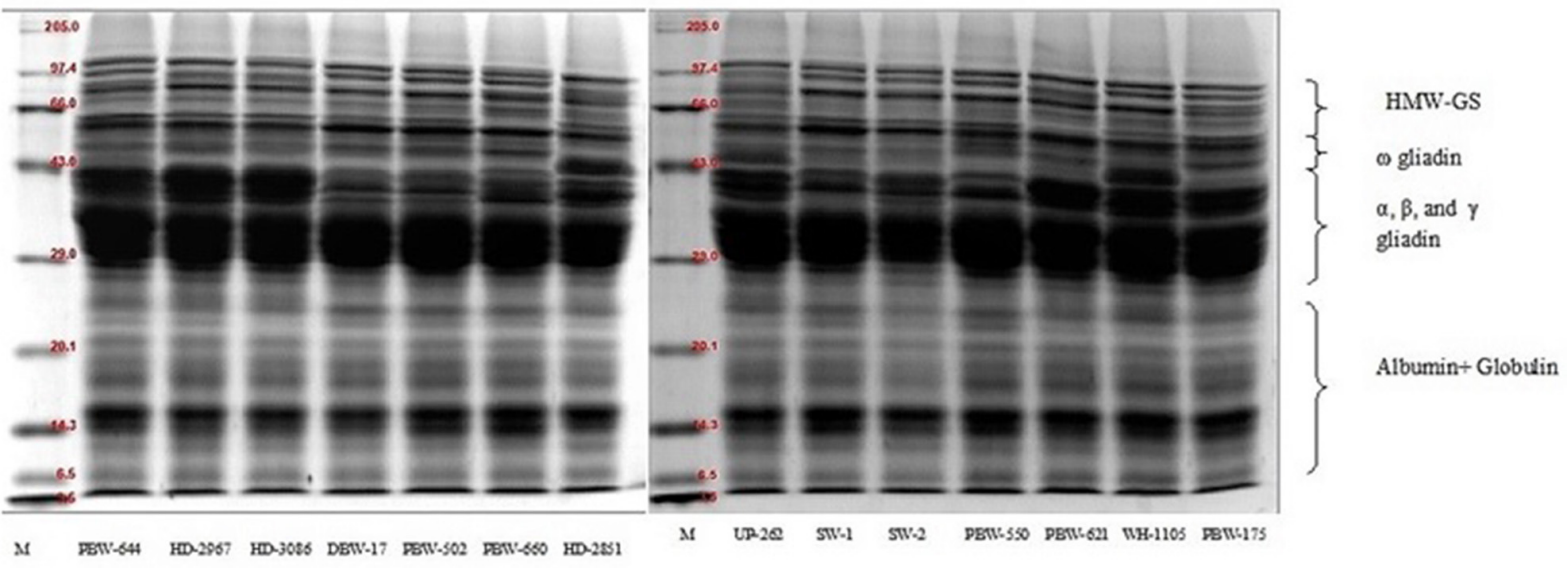
SDS-PAGE patterns of total flour proteins of the different wheat varieties of North India under reducing conditions using 12% resolving gel.

The SDS-PAGE of defatted wheat flour (total flour proteins) from the different wheat cultivars of North India under reducing conditions is presented in Figure 1. The wheat varieties showed the presence of 19–23 polypeptides with a molecular weight range of 4.4–120.8 kDa. The total flour proteins were categorized into four subgroups on the basis of the location of the bands and the molecular mass of the different polypeptides: (i) high-molecular-weight glutenin subunit (HMW-GS; *M*_w_ = 65.1–120.8 kDa); (ii) ω-gliadin (*M*_w_ = 50.7–64.6 kDa); (iii) α-, β-, and γ-gliadin/low-molecular-weight glutenin subunit (LMW-GS; *M*_w_ = 27.1–50.1 kDa); and (iv) A + G (*M*_w_ = 4.4–26.9 kDa). DuPont et al. (53) documented the total flour protein and reported HMW-GS in the molecular weight range 70–112 kDa, ω-gliadin (*M*_w_ = 50–64.6 kDa), α-, β-, and γ-gliadin/LMW-GS (30–45 kDa), and low-molecular-mass albumins in the molecular weight range 6–30 kDa. The variations in the molecular mass between the two studies might be due to the genetic makeup of the cultivars and the growing environment conditions. Each wheat variety showed the presence of four HMW-GS, except SW-1 and SW-2, which resolved into three HMW-GS. Anjum et al. (54) reported that common wheat possesses three to five HMW-GS. The ω-gliadin consisted of two to three polypeptides depending on the variety. All the wheat varieties showed the presence of two ω-gliadin polypeptides, except PBW-502 and PBW-175, which resolved into three bands. High polymorphism both in the number as well as the intensity was observed in the molecular weight range 35.1–42.8 kDa, which corresponds to the α-, β-, and γ-gliadin/LMW-GS region. Low-intensity polypeptide chains around 35.1–43.6 kDa were spotted in the wheat varieties DBW-17, PBW-502, PBW-660, SW-1, and SW-2. The 35.1–43.6-kDa region can thus be used as a genetic biomarker to differentiate wheat varieties (55). The resolution of the protein bands between 26.1–34.5 kDa was not good enough so that the several individual components could be identified. Wheat varieties HD-2851 and WH-1105 both were distinguished by the presence of prominent high-intensity bands around 42.8 and 41.1 kDa, respectively. Irrespective of the wheat variety, the polypeptides in the molecular weight range 4.4–26.9 kDa, which corresponds to the A + G proteins, did not show any significant variation and were almost identical. A little to no polymorphism in both albumin and globulin has also been documented by other authors (56, 57).

The proportions of the different flour proteins determined densitometrically are given in Table 9 and Figure S2. HMW-GS varied from 8.05 to 16.33% of the total extractable flour proteins. Statistically, a significant (*p* ≤ 0.05) difference was observed in HMW-GS among the wheat cultivars. The highest proportion of HMW-GS was observed in the wheat variety PBW-660 and the lowest in SW-1. HMW-GS are minor components and constitute only 5–10% of the total protein, but account for nearly 70% variation in bread quality (58). HMW-GS are mainly responsible for providing elasticity to the dough, allowing the gas produced by the yeast to be trapped and making the products rise during fermentation. Several studies have found a strong positive correlation between the proportion of HMW-GS and the bread-making parameters (12, 59, 60).

**Table 9:** Proportion of total proteins in flours of the different wheat varieties under reducing conditions.

**Variety**	**HMW-GS**	**ω-gliadin**	**α-, β-, γ-gliadin/LMW-GS**	**A + G**	**HMW-GS/LMW-GS**
DBW-17	10.24 ± 0.97^AB^	9.69 ± 4.90^A^	22.30 ± 4.22^A^	57.77 ± 0.29^A^	0.46 ± 0.04^A^
HD-2851	12.22 ± 0.23^AB^	5.79 ± 0.09^A^	26.05 ± 3.00^A^	55.94 ± 2.68^A^	0.47 ± 0.06^A^
HD-2967	12.27 ± 1.14^AB^	7.98 ± 2.70^A^	18.971 ± 0.26^A^	60.78 ± 3.58^A^	0.65 ± 0.07^A^
HD-3086	12.76 ± 1.77^AB^	6.83 ± 0.79^A^	20.37 ± 1.44^A^	60.04 ± 4.00^A^	0.63 ± 0.04^A^
PBW-175	10.87 ± 0.72^AB^	4.47 ± 0.14^A^	28.19 ± 6.34^A^	56.48 ± 5.75^A^	0.40 ± 0.12^A^
PBW-502	15.07 ± 2.64^AB^	11.25 ± 4.43^A^	20.81 ± 6.55^A^	52.87 ± 8.33^A^	0.78 ± 0.37^A^
PBW-550	12.15 ± 3.58^AB^	3.82 ± 0.10^A^	18.76 ± 0.32^A^	65.27 ± 3.16^A^	0.65 ± 0.20^A^
PBW-621	9.138 ± 1.18^B^	9.38 ± 1.98^A^	31.81 ± 5.63^A^	49.67 ± 4.82^A^	0.29 ± 0.01^A^
PBW-644	13.293 ± 1.34^AB^	5.77 ± 0.33^A^	21.14 ± 6.44^A^	59.80 ± 4.77^A^	0.67 ± 0.27^A^
PBW-660	16.33 ± 2.45^A^	13.09 ± 1.80^A^	20.97 ± 2.96^A^	49.61 ± 7.22^A^	0.78 ± 0.01^A^
SW-1	8.05 ± 1.44^B^	9.52 ± 3.27^A^	25.58 ± 1.69^A^	56.85 ± 3.01^A^	0.32 ± 0.08^A^
SW-2	10.07 ± 1.43^AB^	6.67 ± 0.66^A^	18.36 ± 1.32^A^	64.90 ± 0.78^A^	0.55 ± 0.12^A^
UP-262	8.44 ± 1.97^B^	5.97 ± 0.14^A^	25.81 ± 6.29^A^	59.78 ± 8.40^A^	0.33 ± 0.00^A^
WH-1105	10.66 ± 1.52^AB^	4.47 ± 3.64^A^	23.04 ± 8.02^A^	61.83 ± 2.86^A^	0.51 ± 0.24^A^

*Data are presented as the mean ± SD. Means with different superscripts in the column differ significantly (p ≤ 0.05). n = 2*.

*HMW-GS, high-molecular-weight glutenin subunit; LMW-GS, low-molecular-weight glutenin subunit*.

The proportion of ω-gliadin ranged from 4.47 to 13.09% of the total extractable flour proteins. A non-significant (*p* ≥ 0.05) difference was observed in the ω-gliadin proportion among the wheat varieties. The α-, β-, and γ-gliadin/LMW-GS also varied non-significantly (*p* ≥ 0.05) from 18.36 to 31.81% of the total extractable flour proteins. The results are in concordance with those of other authors (60, 61) who found that α-, β-, and γ-gliadin occur in high proportions compared to that of ω-gliadin. Depending on their properties, various gliadin subfractions have been found to be differently associated with dough quality (59). Khatkar et al. (62) found that the addition of α-, β-, γ-, and ω-gliadin resulted in improved bread-making quality; however, the role of ω-gliadin is debatable (59, 63). The percentage of A + G determined after densitometric scanning of the SDS-PAGE gels was the highest among the different fractions and varied non-significantly (*p* ≥ 0.05) from 49.67 to 65.27% of the total flour proteins. Miháliková et al. (64) used SDS-PAGE for the determination of the different flour proteins and obtained HMW-GS (10.35–20.53%), LMW-GS (51.34–76.10%), albumin, and globulin (11.48–34.12%). The results revealed wide variations in the proportions of the different protein types, mainly LMW-GS and A + G. The differences may be because of the genetic makeup, seasonal variation, and the application of nitrogen fertilizers, which influence the balance of various protein fractions (11). The ratio of HMW-GS to LMW-GS ranged from 0.32 to 0.78, but the variation was non-significant (*p* ≥ 0.05). Wheat varieties having a higher HMW-GS/LMW-GS ratio are generally associated with improved rheological and bread-making qualities (12).

### Pearson's Correlation Coefficient

Table S1 summarizes the Pearson's correlation coefficients between the various flour components and wheat grains. Wheat flour protein showed a significant positive relation with wheat kernel brightness (L^*^), yellowness (b^*^), the total color difference (Δ*E*), chroma, and hue. Wang et al. (65) also reported a significant relationship between the protein content of flour and the color parameters. Flour protein content (PC) was positively correlated with wet gluten (*r* = 0.621, *p* ≤ 0.05) and dry gluten (*r* = 0.625, *p* ≤ 0.05), which is expected because the flour protein content is mainly due to gluten proteins. A positive correlation between PC and gluten (both wet and dry) has also been reported earlier (66). PC showed no correlation with the sedimentation value (SV; *r* = 0.042), which emphasized that SV should not be used as a sole criterion for determining protein quality as well as quantity. Panghal et al. (39) also reported a correlation of *r* = 0.091 between PC and SV. No relationship between the protein content and any other SRC values except SCSRC (*r* = −0.598;, *p* ≤ 0.05) was found. Duyvejonck et al. (41) also did not find any significant relationship between the protein content and any of the SRC values. The protein content exhibited non-significant positive relations with HMW-GS (*r* = 0.286), ω-gliadin (*r* = 0.119), and HMW-GS/LMW-GS (*r* = 0.248) and negative relations with LMW-GS/α-, β-, and γ-gliadin (*r* = −0.096) and A + G (*r* = −0.130). Ash content was negatively correlated with L^*^ (*r* = −0.655, *p* ≤ 0.05) and Δ*E* (*r* = −0.672, *p* ≤ 0.01). The results suggest that the lightness of the flour is controlled by the amount of ash, which in turn is directly controlled by bran contamination in flour. A similar relationship between flour brightness and ash content has been reported by Dennett and Trethowan (67).

Wet gluten was positively correlated with SV (*r* = 0.535, *p* ≤ 0.05) and showed a strong positive relation with dry gluten (*r* = 0.983) at the 0.01% level of significance. Gulia and Khatkar (66) also reported a correlation (*r* = 0.92, *p* ≤ 0.01) between wet and dry gluten.

SV showed a significant positive correlation with GPI (*r* = 0.559, *p* ≤ 0.05) and a non-significant positive correlation with LASRC (*r* = 0.303). Flour brightness (L^*^) was negatively related with redness (a^*^), yellowness (b^*^), and chroma, respectively (*r* = −0.595, *r* = −0.585, and *r* = −0.587; *p* ≤ 0.05) and positively with Δ*E* (*r* = 0.989, *p* ≤ 0.01), which reflected that the color parameters of flour are inversely related to each other, unlike color parameters in wheat grains which are positively related. The reason may be that the color in wheat kernels is due to the combination of various pigments which are mostly concentrated in the outer regions and removed during milling; in flour, it is mostly due to ash and bran contamination and only to a small extent by pigments. Similar results were obtained by Wang et al. (65).

a^*^ was negatively correlated with Δ*E* (*r* = −0.560, *p* ≤ 0.05) and hue (*r* = −0.964, *p* ≤ 0.01). WSRC was positively related with SUSRC (*r* = 0.570, *p* ≤ 0.05) and showed a strong positive correlation with SCSRC (*r* = 0.816, *p* ≤ 0.01), which indicated that starch damage and pentosan content are the major factors determining water absorption in flour. WSRC also showed a non-significant positive correlation with LASRC (*r* = 0.472). The positive relation between water and the other SRC values is due to the ability of the water to hydrate and swell up all the major polymeric flour constituents. Similar results were reported by Pasha et al. (44). A positive correlation was observed between SUSRC and LASRC (*r* = 0.565, *p* ≤ 0.05). LASRC showed a highly significant positive correlation with GPI (*r* = 0.791, *p* ≤ 0.01). LASRC, GPI, and SV all reflect protein quality and gluten strength and are based on the swelling capacity of glutenin strands in the lactic acid medium, which might explain the positive relationship between them. A positive correlation between LASRC and SV was also reported by Karaduman (45). The ratio of HMW-GS to LMW-GS was found to be positively related with HMW-GS (*r* = 0.915) and negatively with LMW-GS (*r* = −0.791) at the 0.01% level of significance. A + G was negatively related with ω-gliadin (*r* = −0.558, *p* ≤ 0.05) and LMW-GS/α-, β-, and γ-gliadin (*r* = −0.724, *p* ≤ 0.01). A non-significant negative correlation was observed between PC and all the amino acids except aspartic acid, glutamic acid, and threonine, which showed positive correlations. An increase in PC is usually accompanied by a decrease in essential amino acids, particularly lysine, and has been reported several times (51, 68). The HMW-GS proportions were positively correlated with tyrosine (*r* = 0.535, *p* ≤ 0.05) and negatively related with methionine (*r* = −0.586), phenylalanine (*r* = −0.579), and total essential amino acid (TEAA; *r* = 0.587) at the 0.05% level of significance. HMW-GS also showed a non-significant positive relation with aspartic acid (*r* = 0.180), glutamic acid (*r* = 0.483), alanine (*r* = 0.506), arginine (*r* = 0.416), and total non-essential amino acid (TNEAA; *r* = 0.442) and was negatively correlated with the essential amino acids. The ratio of HMW-GS to LMW-GS was found to be significantly correlated with arginine (*r* = 0.535) and tyrosine (*r* = 0.645). The HMW-GS/LMW-GS ratio also showed a non-significant positive correlation with TNEAA (*r* = 0.327) and a significant negative correlation with TEAA (*r* = −0.444). This reflects the importance of NEAA in dough and baking quality. HMW-GS are rich in tyrosine, and these amino acids, although low in concentrations, are involved in covalent bond formation involving tyrosine–tyrosine crosslinks between glutenins and gliadin (61). These covalent bonds are believed to play an important role in determining the properties and structure of gluten. A negative correlation was observed between ω-gliadin and TEAA (*r* = −0.560, *p* ≤ 0.05). LMW-GS/α-, β-, and γ-gliadin, on the other hand, were positively correlated with the essential amino acids and showed negative relations with the non-essential amino acids. Glutamic acid showed a significant positive correlation with TNEAA (*r* = 0.678, *p* ≤ 0.01), alanine (*r* = 0.684, *p* ≤ 0.01), and tyrosine (*r* = 0.579, *p* ≤ 0.05) and a negative correlation with methionine (*r* = −0.794, *p* ≤ 0.01), which is expected as glutamic acid is the predominant amino acid in wheat flour. A highly significant negative correlation (*r* = −0.719, *p* ≤ 0.01) was observed between cystine and lysine. Cystine is formed by two molecules of cysteine linked together by a disulfide linkage. Cysteine is known to form inter- and intrachain disulfide linkages between various gluten proteins (61), and such formation is believed to play an important role in the dough and baking quality, which might support a negative relation between increasing protein content and lysine. TNEAA was negatively related with TEAA, although non-significantly.

## Conclusion

The study was helpful in understanding the diversity of wheat varieties grown in different geographical regions of North India in terms of their grain, flour, protein profiling, proportions of the different proteins, and the amino acid composition. The study concluded that the wheat varieties showed significant diversity in almost all the quality traits. Moderate protein, gluten content, SDS sedimentation value, and solvent retention capacity suggest that most of these varieties have weak gluten strength more suitable for chapatti and biscuit making. Polymorphism both in the number and intensity of bands was observed particularly in the HMW-GS, ω-gliadin, and the α, β, and γ-gliadin/LMW-GS region. Protein profiling of A + G proteins did not show significant variations and they were almost identical. The wheat varieties HD-2967, HD-3086, PBW-502, PBW-644, and PBW-660, having better protein, gluten, sedimentation volume, proportion of HMW-GS, and higher HMW-GS/LMW-GS ratio, can be further improved for their amino acid composition. Our results could be beneficial to plant breeders, millers, and bakers in selecting those wheat varieties with better quality characteristics for end product use without compromising the nutritional value and thus can also be used for future breeding programs.

## Data Availability Statement

The raw data supporting the conclusions of this article will be made available by the authors, without undue reservation.

## Author Contributions

RS collected the samples, performed the analysis, interpreted and analyzed the data, and wrote the manuscript, with contributions from TS and MR. DS along with RS planned the work, checked the manuscript thoroughly, and made critical revisions before final submission. MB helped in the statistical analysis. All authors read and checked the manuscript properly before submission.

## Conflict of Interest

The authors declare that the research was conducted in the absence of any commercial or financial relationships that could be construed as a potential conflict of interest.
