# Supplementary material for: Diversity in Grain, Flour, Amino Acid Composition, Protein Profiling, and Proportion of Total Flour Proteins of Different Wheat Cultivars of North India
Source: Front Nutr. 2020 Sep 8;7:141. doi: 10.3389/fnut.2020.00141 (PMC7506077; doi:10.3389/fnut.2020.00141)
Supplement: Supplementary file 1 [file Data_Sheet_1.pdf]

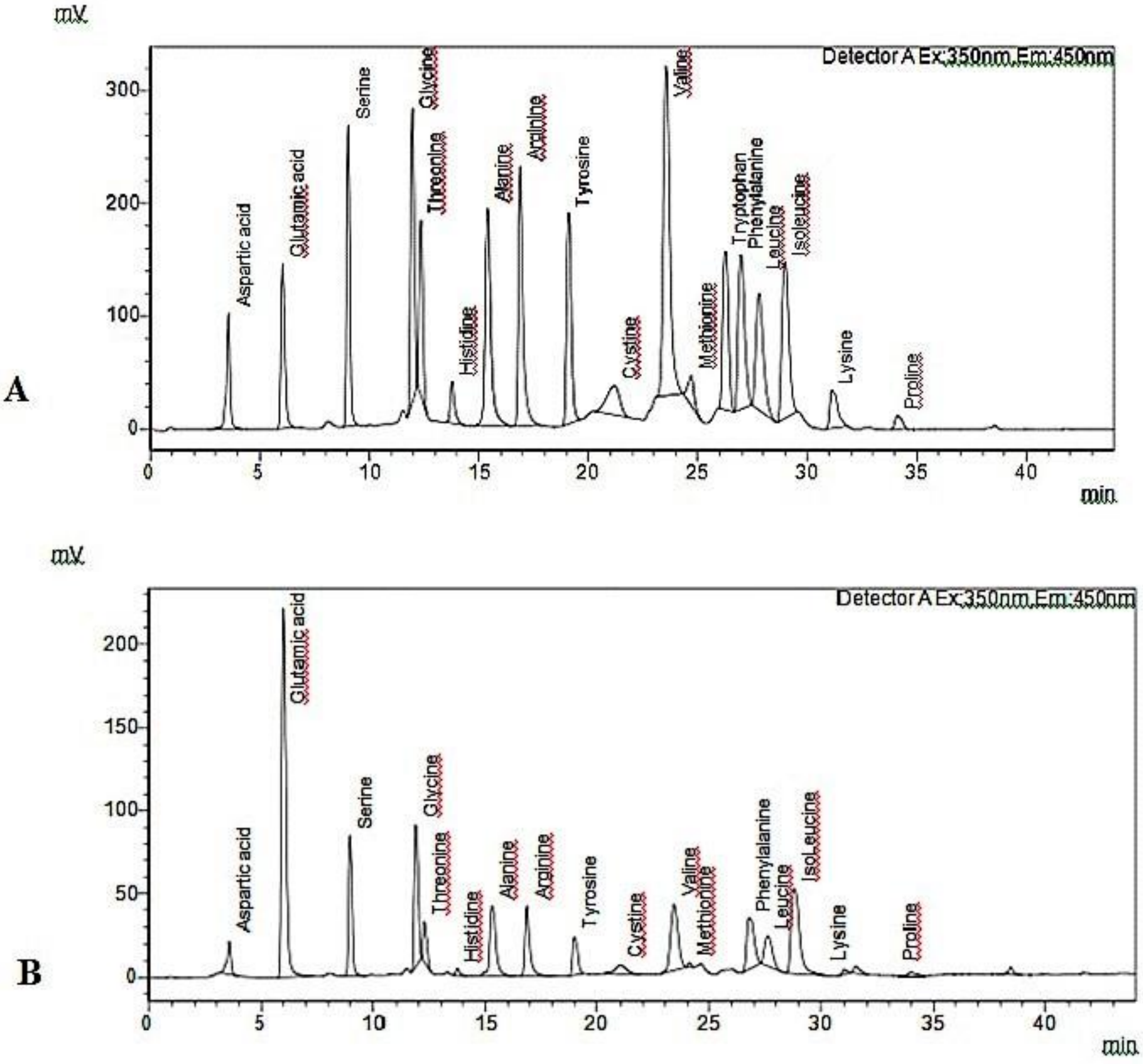

Fig S1| HPLC chromatograms showing (A) Standard mixture of 18 amino acids (B) Amino acid profiling of one of the 14 wheat varieties (HD-2967) . X- axis showing the retention time (min) ; Y- axis intensity in mv.

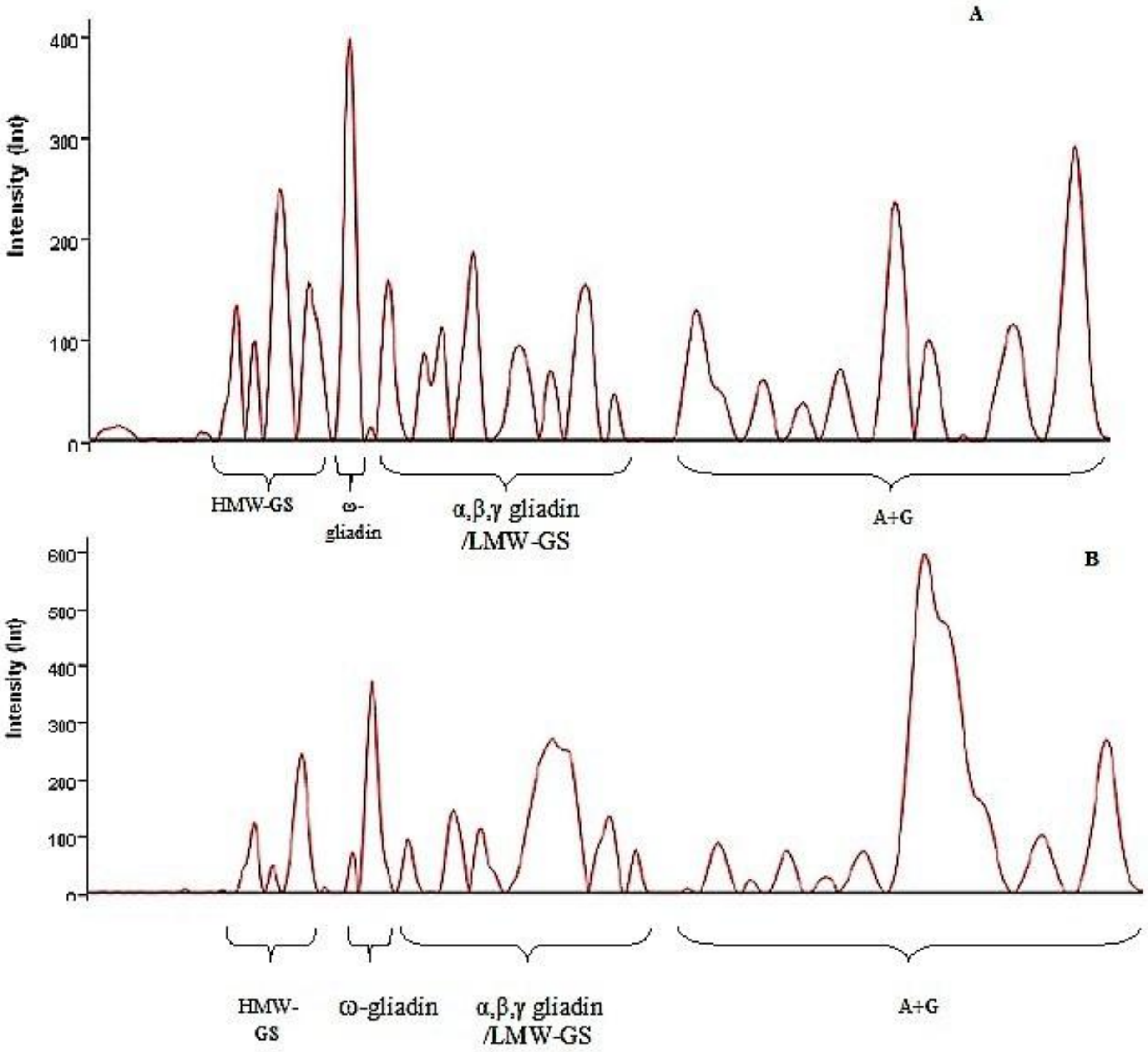

Fig S2| Densitograms illustrating the relative proportion of total flour proteins in different wheat varieties (A) PBW-660 (B) SW-1. The densitograms clearly depicting high proportion of HMW-GS and low proportion of A+G in PBW-660 as compared to SW-1.

Table S1 | Correlation Coefficient between grain and flour quality characteristics, amino acids composition and proportion of different protein fractions.

	L*w	a*w	b*w	ΔEw	Chroma w	Hue w	PC	FC	AC	FiC	WG	DG	SV	L*f	a*f
L*w	1														
a*w	.249	1													
b*w	.813**	.444	1												
ΔEw	.993**	.317	.873**	1											
Cw	.787**	.565*	.990**	.853**	1										
Hw	.334	-.742**	.271	.306	.133	1									
PC	.579*	-.094	.630*	.595*	.564*	.567*	1								
FC	.430	.162	.296	.419	.296	.058	.290	1							
AC	.308	.296	.317	.323	.337	-.083	.347	.137	1						
FiC	.424	-.198	.333	.409	.275	.470	.313	.620*	.143	1					
WG	.428	-.293	.229	.392	.162	.486	.621*	.454	.346	.436	1				
DG	.415	-.314	.280	.388	.205	.548*	.625*	.420	.313	.459	.977**	1			
SV	-.074	-.267	-.164	-.099	-.194	.164	.042	.157	.142	.118	.023	-.034	1		
L*f	-.087	-.045	-.064	-.086	-.066	.012	-.268	.197	-.655*	.017	-.138	-.103	.227	1	
a*f	.494	.196	.497	.510	.491	.157	.424	.127	.258	.418	.199	.174	-.468	-.595*	1
b*f	.056	.465	.184	.095	.245	-.369	-.103	-.280	.296	-.412	-.326	-.388	-.335	-.585*	.479
ΔE f	-.075	.027	-.049	-.070	-.040	-.055	-.322	.171	-.672**	-.049	-.201	-.177	.183	.989**	-.560*
Hue f	-.544*	-.087	-.494	-.548*	-.470	-.273	-.479	-.235	-.187	-.593*	-.325	-.312	.428	.469	-.964**
Chromaf	.061	.467	.187	.099	.248	-.369	-.100	-.275	.298	-.407	-.324	-.386	-.336	-.587*	.485
WSRC	-.024	.472	-.003	-.008	.069	-.519	-.414	-.114	-.027	-.498	-.184	-.152	-.211	.146	-.382
SUSRC	.060	.288	.142	.082	.172	-.203	.063	.160	.422	-.045	.215	.326	-.027	-.217	-.130
LASRC	-.003	.316	.034	.011	.078	-.312	-.175	.374	.142	.248	.113	.158	.303	.287	-.302
SCSRC	-.182	.425	-.110	-.162	-.035	-.545*	-.598*	-.304	-.140	-.398	-.486	-.423	-.341	.133	-.352
GPI	.018	.094	-.009	.016	.005	-.102	-.028	.472	.002	.426	.190	.165	.599*	.411	-.186
HMW-GS	.266	.404	.347	.297	.381	-.163	.286	.551*	.020	.056	.043	.084	-.041	.314	-.197
ω-gliadin	.144	.772**	.470	.227	.555*	-.473	.119	.059	.230	-.230	-.341	-.378	-.134	.034	.197
LMW-GS	-.271	-.239	-.267	-.281	-.283	.060	-.096	-.320	-.157	-.144	.075	.090	-.316	-.015	.167
AG	.009	-.442	-.219	-.046	-.272	.300	-.130	-.043	-.013	.220	.111	.100	.353	-.161	-.151
HMW-GS/LMW-GS	.389	.394	.416	.411	.443	-.105	.248	.568*	.110	.169	.042	.063	.124	.264	-.198

	b*f	ΔE f	Hue f	Chroma f	WSRC	SUSRC	LASRC	SCSRC	GPI	HMW-GS	ω-gliadin	LMW-GS	AG	HMW-GS/LMW-GS
L*w														
a*w														
b*w														
ΔEw														
Cw														
Hw														
PC														
FC														
AC														
FiC														
WG														
DG														
SV														
L*f														
a*f														
b*f	1													
ΔE f	-.466	1												
Hue f	-.233	.462	1											
Chroma f	1.000**	-.468	-.240	1										
WSRC	.193	.200	.452	.190	1									
SUSRC	-.075	-.254	.127	-.074	.570*	1								
LASRC	-.365	.251	.211	-.364	.472	.565*	1							
SCSRC	.093	.166	.395	.090	.816**	.429	.303	1						
GPI	-.433	.375	.067	-.431	-.016	.026	.791**	-.217	1					
HMW-GS	-.318	.277	.138	-.316	.111	.332	.179	.137	.030	1				
ω-gliadin	.484	.103	-.065	.485	.029	-.100	-.081	.006	-.032	.343	1			
LMW-GS	.112	.007	-.147	.112	-.202	-.085	-.196	-.317	-.063	-.505	-.015	1		
AG	-.210	-.200	.088	-.212	.094	-.036	.118	.188	.054	-.274	-.724**	-.558*	1	
HMW-GS/LMW-GS	-.297	.229	.135	-.296	.161	.213	.233	.222	.082	.915**	.248	-.791**	.055	1

w= wheat, f= flour, PC = protein content, FC = fat content, AC= ash content, FiC = fibre content, WG = wet gluten, DG= dry gluten, SV= SDS-Sedimentation Volume, WSRC, SUSRC, LASRC, SCSRC = water, sucrose, lactic acid, sodium carbonate solvent retention capacity, GPI= Gluten Performance Index, HMW-GS = High molecular weight glutenin subunits, LMW-GS = low molecular weight glutenin subunits, AG = Albumin+Globulin

* Significant at 0.05 level ; ** Significant at 0.01 level

	PC	WG	DG	SV	HMWGS	ω-gliadin	LMWGS	AG	HMW//LMW	Asp	Glu	Ser	Gly
PC													
WG													
DG													
SV													
HMWGS													
ω-gliadin													
LMWGS													
AG													
HMW//LMW									1				
Asp	.410	.199	.249	-.277	.180	.220	-.148	-.093	.144	1			
Glu	.120	.192	.321	-.316	.483	-.060	-.071	-.145	.380	.196	1		
Ser	-.207	.288	.244	.530	-.233	-.506	-.099	.483	-.096	-.355	.160	1	
Gly	-.498	-.059	-.069	-.006	-.235	-.470	-.247	.584*	-.024	-.497	-.151	.392	1
Ala	-.090	-.051	.028	-.203	.506	.186	-.098	-.274	.386	-.003	.684**	.215	-.018
Arg	-.278	-.326	-.320	-.028	.416	.230	-.556*	.118	.535*	-.078	.318	.280	.423
Tyr	-.037	-.138	-.109	-.167	.535*	.110	-.589*	.155	.645*	.062	.579*	.154	.234
Cys-Cys	.065	-.038	-.103	.093	-.241	.375	-.108	-.007	-.155	.539*	-.172	.031	-.398
Pro	-.042	-.028	-.021	-.035	-.092	-.291	.303	-.037	-.210	-.320	-.420	-.411	-.009
T NEAA	-.047	.123	.252	-.398	.442	-.259	-.109	.020	.327	.204	.678**	-.056	-.034
Thre	.089	.465	.401	-.044	-.448	-.059	.343	-.026	-.437	.231	-.047	.391	.022
His	-.101	.084	.096	-.269	-.150	.118	.049	-.033	-.113	.283	.394	.109	-.111
Val	-.281	.272	.315	.043	-.215	-.145	.258	-.023	-.200	.030	.361	.380	.093
Met	-.031	-.040	-.168	.142	-.586*	-.193	.177	.254	-.522	-.325	-.794**	.036	.260
Phe	-.103	.288	.230	.036	-.579*	-.447	.043	.504	-.447	.261	-.215	.369	.210
Ile	-.205	-.080	-.060	.033	-.342	-.418	-.140	.520	-.206	.179	.227	.426	.192
Leu	-.103	-.073	-.033	.272	-.157	-.504	-.190	.518	-.012	-.324	.337	.448	.205
Lys	-.171	-.234	-.248	.090	.227	-.008	.036	-.136	.163	-.478	-.399	-.352	.223
TEAA	-.297	.138	.101	.184	-.587*	-.560*	.120	.508	-.444	-.207	-.107	.481	.383

	Ala	Arg	Tyr	Cys-Cys	Pro	T NEAA	Thre	His	Val	Met	Phe	Ile	Leu	Lys	TEAA
PC															
WG															
DG															
SV															
HMW-GS															
ω-gliadin															
LMW-GS															
AG															
Asp															
Glu															
Ser															
Gly															
Ala	1														
Arg	.599*	1													
Tyr	.399	.802**	1												
Cys-Cys	.009	.007	-.140	1											
Pro	-.382	-.654*	-.635*	-.455	1										
T NEAA	.651*	.109	.181	-.127	.118	1									
Thre	-.185	-.047	-.025	.366	-.485	-.368	1								
His	.035	.092	.347	.438	-.532	.088	.551*	1							
Val	.077	-.111	-.053	.003	-.286	.069	.361	.352	1						
Met	-.569*	-.294	-.451	.099	.368	-.517	.171	-.184	-.503	1					
Phe	-.435	-.179	-.091	.423	-.204	-.173	.696**	.541*	.154	.435	1				
Ile	-.162	.193	.418	.204	-.480	-.049	.433	.618*	.144	.053	.715**	1			
Leu	-.138	.059	.395	-.257	-.165	-.005	-.039	.326	.140	-.043	.233	.696**	1		
Lys	-.299	-.142	-.178	-.719**	.693**	-.185	-.497	-.661*	-.245	.154	-.493	-.536*	-.177	1	
TEAA	-.531	-.188	.031	-.051	-.094	-.311	.523	.490	.360	.325	.768**	.747**	.647*	-.169	1

PC = protein content, WG = wet gluten, DG= dry gluten, SV= SDS-Sedimentation Volume, , HMW-GS = High molecular weight glutenin subunits, LMW-GS = low molecular weight glutenin subunits, AG = Albumin+Globulin, HMW/LMW= Ratio HMW-GS/LMW-GS, Asp= Aspartic acid, Glu = Glutamic acid , Ser = Serine, Gly= Glycine, Ala = Alanine, Arg = Arginine, Tyr = Tyrosine, Cys-Cys = Cystine, Pro= Proline, T NEAA = Total non-essential amino acids, Thre = Threonine, His = Histidine , Val = Valine, Met = Methionine, Phe = Phenyalanine, Ile = Isoleucine, Leu = leucine, Lys = Lysine, TEAA = Total essential amino acids.

* Significant at 0.05 level ; ** Significant at 0.01 level.
